# Safety and Efficacy of Nucleic Acid Polymers in Monotherapy and Combined with Immunotherapy in Treatment-Naive Bangladeshi Patients with HBeAg+ Chronic Hepatitis B Infection

**DOI:** 10.1371/journal.pone.0156667

**Published:** 2016-06-03

**Authors:** Mamun Al-Mahtab, Michel Bazinet, Andrew Vaillant

**Affiliations:** 1 Bangabandhu Sheikh Mujib Medical University, Shahbagh Road, Dhaka-1000, Bangladesh; 2 Replicor Inc., 6100 Royalmount Avenue, Montreal, Quebec, Canada H4P 2R2; Taipei Veterans General Hosptial, TAIWAN

## Abstract

Previous *in vivo* studies have suggested that nucleic acid polymers (NAPs) may reduce circulating levels of HBsAg in the blood by blocking its release from infected hepatocytes and that this effect may have clinical benefit. NAP treatment, was evaluated in two clinical studies in patients with HBeAg positive chronic HBV infection. The REP 101 study examined REP 2055 monotherapy in 8 patients and the REP 102 study examined REP 2139-Ca, in monotherapy in 12 patients, 9 of which transitioned to short term combined treatment with pegylated interferon alpha 2a or thymosin alpha 1. In both studies NAP monotherapy was accompanied by 2–7 log reductions of serum HBsAg, 3–9 log reductions in serum HBV DNA and the appearance of serum anti-HBsAg antibodies (10–1712 mIU / ml). Eight of the 9 patients transitioning to combined treatment with immunotherapy (pegylated interferon or thymosin alpha 1) in the REP 102 study experienced HBsAg loss and all 9 patients experienced substantial increases in serum anti-HBsAg antibody titers before withdrawal of therapy. For 52 weeks after removal of REP 2055 therapy, rebound of serum viremia (HBV DNA > 1000 copies / ml, HBsAg > 1IU / ml) was not observed in 3 / 8 patients. Suppression of serum virema was further maintained for 290 and 231 weeks in 2 of these patients. After withdrawal of all therapy in the 9 patients that transitioned to combination therapy in the REP 102 study, 8 patients achieved HBV DNA < 116 copies / ml after treatment withdrawal. Viral rebound occurred over a period of 12 to 123 weeks in 7 patients but was still absent in two patients at 135 and 137 weeks of follow-up. Administration tolerability issues observed with REP 2055 were rare with REP 2139-Ca but REP 2139-Ca therapy was accompanied by hair loss, dysphagia and dysgeusia which were considered related to heavy metal exposure endemic at the trial site. These preliminary studies suggest that NAP can elicit important antiviral responses during treatment which may improve the effect of immunotherapy. NAPs may be a potentially useful component of future combination therapies for the treatment of chronic hepatitis B.

***Trial Registration*:** ClinicalTrials.gov NCT02646163 and NCT02646189

## Introduction

Chronic hepatitis B virus (HBV) is an infection of the liver that affects more than 260 million people worldwide [1]. With increasing duration of infection, HBV increases the risk of liver cirrhosis and hepatocellular carcinoma. HBV-infected hepatocytes produce two types of viral particles: infectious virus (Dane particles) and non-infectious subviral particles (SVPs). Although both virus and SVPs are coated with the hepatitis B surface antigen (HBsAg), SVPs are present in 1,000–100,000 fold excess over infectious virus [2] and constitute the bulk of HBsAg present in the circulation. The clearance of HBsAg from the blood and concomitant appearance of free anti-HBsAg antibodies (anti-HBs) is widely accepted to be the best prognostic indicator for achieving durable control of chronic HBV infection (CHB) either spontaneously or as a result of treatment [3, 4], suggesting HBsAg plays an important role in the maintenance of CHB. Currently approved treatments for CHB include interferon-based and thymosin α1 immunotherapies and nucleos(t)ide HBV polymerase inhibitors. Treatment with pegylated interferons can achieve a durable control of HBV infection with 48 weeks of therapy but only in a small fraction of patients [5]. Treatment with HBV polymerase inhibitors like entecavir (ETV) and tenofovir disoproxil fumarate (TDF) suppress the levels of infectious virus in the blood [6–8] but have little effect on serum HBsAg [9] which may be in part responsible for the requirement of long term chronic treatment to maintain control of serum viremia [10]. As such, there is still a need for more effective treatments for CHB which target the reduction of HBsAg in the blood.

Nucleic acid polymers (NAPs) have both entry and post-entry antiviral activity in duck hepatitis B virus (DHBV) infected Pekin duck hepatocytes *in vitro* and *in vivo* [11, 12]. The NAP REP 2055was optimized for activity and tolerability in DHBV infected ducks and was an effective prophylactic agent for preventing DHBV infection, an effect shown to be dependent on a non-immunostimulatory, post-entry antiviral activity [11, 12]. In the therapeutic setting, REP 2055 treatment in established DHBV infection resulted in the rapid clearance of duck HBsAg (DHBsAg) and concomitantly increased titers of anti-DHBsAg antibodies in all ducks [13]. Despite elimination of DHBsAg from the blood, DHBsAg was still found in the liver, suggesting that NAPs block the secretion of DHBsAg. Moreover the persistence of significant serum DHBV DNA in many ducks during treatment despite the absence of detectable serum DHBsAg suggested a selective effect of NAPs on subviral particle secretion from infected hepatocytes [13]. Importantly, the clearance of DHBsAg was associated with the control of DHBV infection for 16 weeks after REP 2055 therapy was discontinued in 55% (6/11) of treated ducks: no evidence of viral antigens (DHBsAg and DHBV core antigen) were found in the liver and covalently closed circular DNA (cccDNA) became transcriptionally inactivated and reduced in copy number by over 200 fold (~2.3 log) compared to the cccDNA copy number in normal saline treated control animals [13].

A small proof of concept trial (REP 101 study) with REP 2055 monotherapy was initiated in Bangladeshi patients with HBeAg positive chronic HBV infection. This trial assessed the safety and efffiacy of REP 2055. With the exception of administration tolerability issues, REP 2055 therapy was generally safe and was accompanied by substantial reductions in serum HBsAg, HBV DNA and the appearance of anti-HBsAg antibodies. To address administration tolerability issues with REP 2055 observed in REP 101 study, a modified version of REP 2055 (REP 2139), was designed and prepared in a novel calcium chelate complex formulation (REP 2139-Ca). A second proof of concept trial (REP 102 study) was conducted in patients with HBeAg+ chronic HBV infection. The primary aims of the REP 102 study were to demonstrate improved administration tolerability, and similar overall antiviral effect of REP 2139-Ca compared to REP 2055 and subsequently, the safety and efficacy of REP 2139-Ca when used in combination with thymosin alpha 1 and or pegylated interferon.

## Materials and Methods

### Study patients and study site

Prospective Bangledeshi patients were screened at the Farabi General Hospital (Dhaka, Bangladesh) and were either current patients at the hospital or referred to the hospital for purposes of trial enrollment. The lengthy weekly dosing regimens involved and the logistical and patient compliance issues in the locale required offering all patients access to therapy in order to enable recruitment. Recruitment for both studies was initiated 2–3 months prior to the planned start of each trial. Treatment naïve subjects were eligible for enrollment in the REP 101 study (REP 2055 treatment) or REP 102 study (REP 2139-Ca treatment) who were between 18 and 55 years of age with body weight < 100kg with a previously documented chronic HBV infection defined as follows: 1) serum HBsAg+, serum anti-HBs < 5 mIU / ml, serum HBV DNA > 10^6^ copies / ml, 2) evidence of liver fibrosis as determined by liver biopsy or Fibroscan analysis, 3) no detectable HIV, HCV or CMV co-infection and 4) the absence of any other co-existent liver disease including NAFLD, autoimmune hepatitis or Wilson’s disease. All patients were serum HBeAg+ except REP 101 patient 8 and all patients had normal liver and kidney function. Patients with liver cirrhosis or any evidence of ascites, hepatic encephalopathy, variceal hemorrhage, cardiac disease, uncontrolled hypertension, diabetes or hematological dysfunction were excluded. Individual pre-treatment baseline characteristics of patients are listed in Table 1. Additional pre-treatment biopsy details for patients in the REP 101 study are provided in S1 Table. All treatments, and sampling were performed on an outpatient basis at the Farabi General Hospital by qualified personnel under supervision of the Principal Investigator. All assessments were performed by the Principal investigator or assistant physicians under the supervision of the Principal Investigator. No incentive was given to any patient other than compensation for travel to the clinic for trial activities and offering all patients whose infection did not respond to treatment or rebounded after treatment access to local generic ETV or TDV for a minimum of 5 years.

**Table 1:** Pretreatment status of Bangladeshi patients enrolled in REP 101 and 102 studies.

Study	Patient (sex)	Age at start of treatment (years)	Confirmed maternal infection ^b^	Genotype ^d^	HBV DNA (cpm)	HBcAg IgM ^d^	HBsAg (qual.)	HBeAg (qual.)	ALT ^g^ (IU / L)	Liver fibrosis score (metavir ^h^)
REP 101	1 (M)	26	No ^c1^	ND	2.1 x10^6^	ND	positive	positive	113	F3
	2(M)	35	Yes	ND	1.3 x 10^7^	ND	positive	positive	39	F2
	3(M)	32	Yes	ND	2.0 x 10^7^	ND	positive	positive	153	F2
	4 (M)	27	No ^c2^	ND	2.3 x 10^11^	ND	positive	positive	84	F1
	5(M)	26	No ^c2^	ND	2.3 x 10^9^	ND	positive	positive	52	F2
	6 (M)	22	No ^c2^	ND	5.0 x 10^11^	ND	positive	positive	58	F2
	7 (M)^a^	25	No ^c2^	D	1.7 x 10^11^	ND	positive	positive	68	F1
	8 (F)	22	Yes	ND	5.0 x 10^6^	ND	positive	negative ^e^	48	F2
REP 102	1(M)	25	Yes	C	>9.89x10^8^	negative	positive	positive	35	F0-F1
	2 (M)	28	No ^c1^	D	>9.89x10^8^	negative	positive	positive	114	F3
	3 (F)	26	No ^c3^	C	1.6x10^8^	negative	positive	positive	52	F0-F1
	4 (M)	20	Yes	A	2.5x10^8^	negative	positive	positive	89	F2
	5 (M)	27	Yes	D	4.5x10^7^	negative	positive	positive	50	F2
	6 (M)	19	Yes	C	1.7x10^8^	negative	positive	positive	124	F0-F1
	7 (M)	22	No ^c3^	C	>9.89x10^8^	negative	positive	positive	116	F0-F1
	8 (F)	22	Yes	D	>9.89x10^8^	negative	positive	positive	41	F0-F1
	9 (M)^a^	26	see above ^a^	D	7.1x10^5^	negative	positive	positive ^f^	21	F1
	10 (M)	18	Yes	C	>9.89x10^8^	negative	positive	positive	36	F0-F1
	11 (M)	18	No ^c3^	C	>9.89x10^8^	negative	positive	positive	70	F0-F1
	12 (M)	26	Yes	C	9.9x10^6^	negative	positive	positive	87	F0-F1

ND = not determined due to sample exhaustion.

^a^ Patient 7 from the REP 101 study was re-enrolled as patient 9 in the REP 102 study (see results).

^b^ maternal transmission of HBV infection confirmed from analysis of individual patient case histories where possible.

^c1^ earliest available HBsAg positive test > 6 months prior to treatment.

^c2^ earliest available HBsAg positive test > 12 months prior to treatment.

^c3^ earliest available HBsAg positive test > 24 months prior to treatment.

^d^ assessed retroactively from frozen serum samples and in some cases could not be determined (ND) due to sample exhaustion. All HBcAg IgM negative samples were confirmed positive for total HBcAg antibodies.

^e^ this HBeAg negative patient was allowed to participate in the REP 101 study on a compassionate basis (see Table 2).

^f^ this patient did not experience HBeAg seroconversion during REP 2055 therapy in the REP 101 study.

^g^ upper limit of normal = 42 IU / ml.

^h^ Metavir score was determined from liver biopsy in REP 101 patients and from Fibroscan analysis in REP 102 patients.

### REP 101 study design

**Fig 1:**
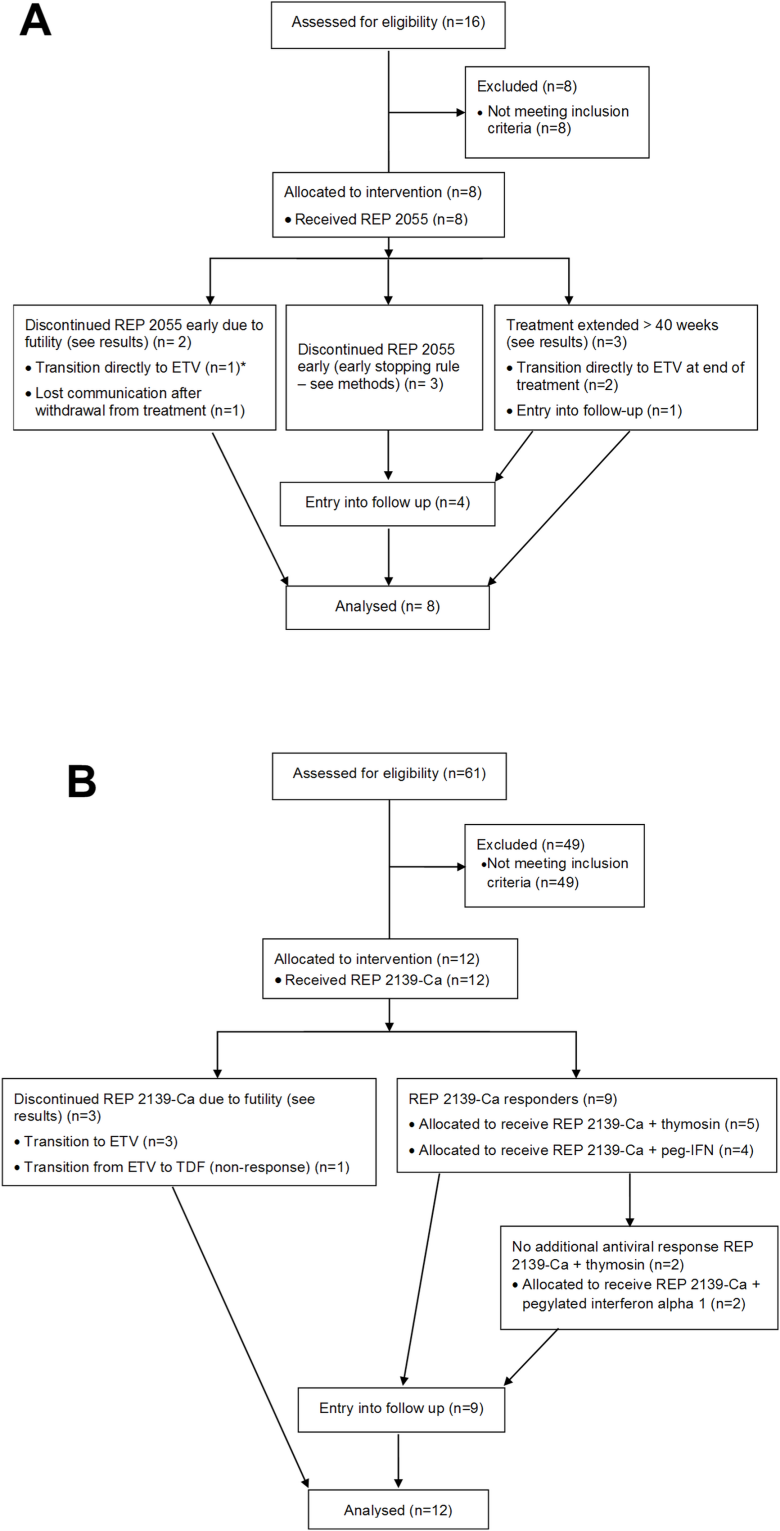
Flow diagrams for the REP 101 study (A) and REP 102 study (B) indicating enrollment, assignment and allocation to treatment, entry into follow-up and analysis. * received 28 weeks of REP 2055 + ETV after REP 2055 monotherapy (patient 8, see methods and results).

The REP 101 study was an open label, non-randomized study. Its primary endpoint was to demonstrate that REP 2055 was well tolerated when given intravenously to patients infected with chronic hepatitis B infection. Its secondary endpoint was to demonstrate an antiviral response in patients receiving REP 2055. Treatment was scheduled for 40 weeks and consisted of dose escalation (100–1200 mg qW) in the first two patients followed by a safety assessment prior to the initiation of dosing in the subsequent 6 patients. These patients received REP 2005 in 400mg doses, based on the minimum efficacious dose observed in the first two patients. Subjects on treatment were eligible for early withdrawal from REP 2055 therapy who experienced the following antiviral response: no detectable serum HBsAg or HBeAg, appearance of anti-HBs > 10 mIU / ml and anti-HBeAg antibodies (anti-HBe) and serum HBV DNA = < 116 copies/ml. After removal of REP 2055 therapy, initial follow-up evaluations were typically every two weeks for the first two months followed by monthly evaluations for the next 4 months followed by visits every 4–6 months. Patients not meeting the criterion for early withdrawal as defined above by the end of treatment or whose viral infection rebounded (HBV DNA > 10^4^ copies / ml) after REP 2055 treatment was withdrawn were offered therapy with locally sourced generic ETV. The REP 101 study flow diagram is provided in Fig 1A. Deviations from the REP 101 protocol were jointly agreed to by the study authors and consented to orally by patients and are reported in Table 2. These deviations were taken in the best interests of providing patients with the best chance to establish control of their infection without undue risk and to be compliant with the declaration of Helsinki.

**Table 2:** REP 101 protocol deviations.

Deviation type	Patient	Description
Exception to exclusion criteria	8	This patient 8 was HBeAg negative but was enrolled on a compassionate basis.
Modification of treatment regimen	2	REP 2055 treatment was restarted in this patient (see methods).
Modification of treatment regimen	2, 4 and 7	Extension of REP 2055 monotherapy permitted beyond 40 weeks.
Modification of follow-up period	1, 3 and 6	Extension of follow-up beyond 1 year
Concomitant therapy*	2	Combined exposure to low dose peg-IFN (18 and 45 ug over two weeks) was attempted during weeks 37 and 38 of the second course of REP 2055 treatment.
Concomitant therapy*	8	Combined exposure to ETV (PO qD 0.5 mg) from treatment weeks 29–56.
Access to standard of care	All	5 years of treatment with local generic ETV for a 5 years was offered to any patient with persistent viremia at the end of treatment or who experienced a rebound in viremia after treatment withdrawal.

* These exposures to concomitant therapy do not affect the outcomes reported in this study.

### REP 102 study design

The REP 102 study was an open label, non-randomized study. Its primary endpoint was to demonstrate that REP 2139-Ca was well tolerated when given intravenously to patients infected with chronic hepatitis B infection in monotherapy and in combination with immunotherapy. Its secondary endpoint was to demonstrate an antiviral response in patients receiving REP 2139-Ca during monotherapy and during combination therapy with REP 2139-Ca and immunotherapy. REP 2139-Ca exposure was scheduled for 40 weeks of 500mg weekly IV infusions (an equivalent molar dose to 400mg of REP 2055) and was initiated after on-treatment safety and efficacy assessments of REP 2055 treatment in the REP 101 protocol were completed. Following interim efficacy and safety analysis, the REP 102 protocol was amended to allow study patients completing >20 weeks of REP 2139-Ca monotherapy with no serious adverse events with HBV DNA > 2000 copies / ml to transition to combination treatment for 13 weeks with thymosin alpha 1 (thymosin; as Zadaxin®) or pegylated interferon alpha 2a (peg-IFN; as Pegasys®). Follow-up assessments and transition to ETV or TDF in the event of viral rebound after withdrawal of therapy with REP 2139-Ca / immunotherapy were as described in the REP 101 study. The REP 102 study flow diagram is provided in Fig 1B. Deviations in the REP 102 protocol were jointly agreed to by the study authors and consented to orally by patients and are reported in Table 3. These deviations were taken in the best interests of providing patients with the best chance to effectively treat their infection without undue risk and to be compliant with the declaration of Helsinki.

**Table 3:** REP 102 protocol deviations.

Deviation type	Patient	Description
Modification to exclusion criteria	All	Patients were required to be HBeAg positive to match the patient population in the REP 101 study.
Exception to exclusion criteria	9	REP 101 patient 7 was allowed to enter the REP 102 trial as patient 9
Modification of treatment regimen	All	Restricting patients with a < 2 log reduction in serum HBV DNA and HBsAg from entering into combination treatment with immunotherapy
Modification of treatment regimen	9, 10, 11 and 13	Allowing exposure to peg-IFN without prior exposure to thymosin in patients
Modification of treatment regimen*	9	Allowing continuation of peg-IFN after halting of REP 2139-Ca in patient 9 to complete 48 weeks of total PEG-IFN exposure (viral rebound occurred after removal of REP 2139-Ca therapy)
Modification of treatment regimen	2,3,4,6,7,8,11 and 12	Entering patients receiving combination therapy into follow-up after 13 / 26 weeks of immunotherapy with the appearance of anti-HBs significantly elevated above 10 mIU / ml
Modification of follow-up period	2, 3, 4 and 11	Extension of follow-up beyond 1 year
Access to standard of care	All	5 years of treatment with local generic ETV for a 5 years was offered to any patient with persistent viremia at the end of treatment or who experienced a rebound in viremia after treatment withdrawal.

* Not shown and does not affect the outcomes reported in this study.

### Study oversight and analysis

All procedures followed in the REP 101 and REP 102 studies were in accordance with the ethical standards of the responsible local institutional ethics committee on human experimentation in Dhaka, Bangladesh (Viral Hepatitis Foundation of Bangladesh) and with the Helsinki Declaration of 1975, as revised in 2008. The REP 101 and REP 102 study protocols and amendments were approved by the Viral Hepatitis Foundation of Bangladesh. All study participants provided written informed consent prior to receiving treatment in the REP 101 and REP 102 studies. The REP 101 (NCT02646163—https://clinicaltrials.gov/ct2/show/NCT02646163) and REP 102 (NCT02646189—https://clinicaltrials.gov/ct2/show/NCT02646189) studies were registered retroactively on www.clincaltrials.gov prior to publication as trial registration in a public database was not a requirement for the conduct of clinical trials in Bangladesh at the time these studies were initiated. Safety and efficacy analyses of patients in the REP 101 and REP 102 studies was performed on an intent to treat basis (Fig 1).

### Study drugs

**Fig 2:**
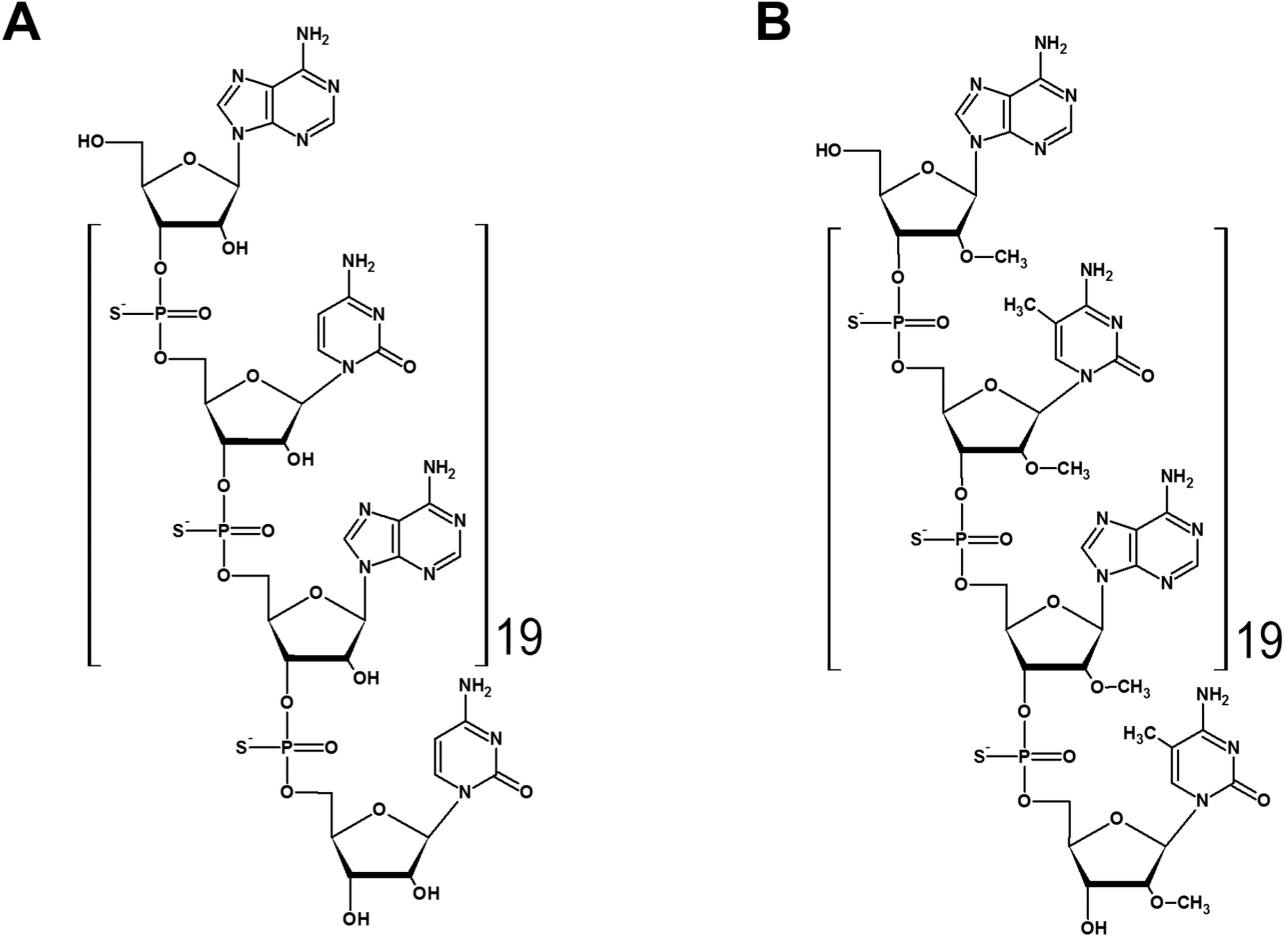
Chemical structures of REP 2055 (A) and REP 2139 (B).

REP 2055 is a phosphorothioate oligodeoxyribonucleotide with the sequence (dAdC)_20_ (Fig 2A). The sodium salt of REP 2055 was synthesized under cGMP and fill finished as a 200mg / ml sterile solution in normal saline in borosilicate vials.

REP 2139 is a phosphorothioate oligoribonucleotide with the same length and sequence as REP 2055 but additionally includes the addition of an O-linked methyl group at the 2’ position of each ribose sugar and the addition of a methyl group at the 5’position of each cytidine base (Fig 2B). The sodium salt of REP 2139 was synthesized under cGMP and fill finished as a sterile 25mg / ml calcium chelate complex solution (REP 2139-Ca) in normal saline in prefilled syringes.

Dosing of both NAPs was controlled by pre-injecting the required dose of REP 2055 or REP 2139-Ca into an IV bag of normal saline with several inversions to ensure mixing prior to the start of administration. Administration was accomplished by slow intravenous (IV) infusion (see results).

### Clinical safety evaluations

While on treatment, all patients underwent weekly safety evaluations which included a physical exam and clinical assessment. This was accompanied each week or every two weeks by assessment of liver and kidney function, hematology and electrolytic status. Both safety and efficacy testing (see below) were performed using blood drawn just prior to the IV infusion of REP 2055 or REP 2139-Ca. The presence of protein, heme or sugar in urine harvested prior to REP 2055 or REP 2139-Ca infusion was monitored by dipstick every visit. A complete biochemical analysis and ECG testing was performed every 4 weeks. Unless warranted by the presentation of symptoms during the clinical assessment, patients coming to the end of the treatment with no blood test abnormalities did not receive additional safety evaluations during the follow-up.

### Clinical efficacy evaluations

#### REP 101 protocol

In the REP 101 study, onsite monitoring of HBsAg clearance was monitored using a local qualitative ELISA (LABAID Diagnostic, Dhaka, Bangladesh). Quantitative serum HBsAg and qualitative serum HBeAg and anti-HBeAg antibody determination was conducted on selected frozen serum samples using the IMPACT platform due to a lack of availability of these test platforms at the trial site. Quantitative assessment of anti-HBsAg antibodies (anti-HBs) was performed in fresh serum samples using the Immulite® ELISA assay (Diagnostic Products Corp) and validated (data not shown) on selected frozen serum samples using the IMPACT platform (Roche Diagnostics, Germany). The quantitative serum HBsAg, anti-HBs and qualitative HBeAg and anti-HBe results were validated (data not shown) for patients 1, 2 and 3 at the South Australia Pathology Unit, Royal Australia Hospital, Adelaide, South Australia in sister frozen serum aliquots using Abbott Architect® quantitative platform.

Quantitative serum HBV DNA was determined in fresh serum samples using the HBV Real-TM Quant PCR assay (Serace Biotechnologies srl., Italy) and validated in selected frozen serum samples from all patients (data not shown) using the Roche cobas® platform (Covance Inc, Germany).

Virology testing after withdrawal of treatment was transitioned to Dr. Lal Path Labs, a Mumbai, India-based diagnostic lab accredited by the College of American Pathologists and the National Accreditation Board for Testing and Validation Laboratories (government of India) and consisted of quantitative determination of serum HBsAg and anti-HBs (Abbott Architect) and quantitative determination HBV DNA (Roche cobas®) in fresh serum samples.

#### REP 102 protocol

Onsite virologic assessment consisted of quantitative determination of serum HBsAg and anti-HBs (Abbott Architect), qualitative determination of HBeAg and anti-HBe (Abbott Architect) and quantitative determination HBV DNA (Roche cobas®) in fresh serum samples at Dr. Lal Path Labs. Quantitative serum HBsAg values obtained at Dr. Lal Path Labs were validated (data not shown) in selected frozen serum samples using the Abbott Architect quantitative HBsAg test platform at the University of Duisburg-Essen (Essen, Germany). HBV genotyping was performed on pre-treatment frozen serum samples at Dr. Lal Path Labs and HBcAg IgM testing (Abbott Architect) was performed on frozen serum samples at pre-treatment or within the first three weeks of treatment at the Institute of Virology, Technische Universität München, Munich, Germany.

## Results

### REP 101 protocol (REP 2055)

#### Dosing, safety and tolerability

Patients 1 and 2 underwent weekly IV dosing of 2055 which escalated from 100mg / week up to 1200mg / week at the end of treatment (23 weeks). Patient 2 in the first cohort was erroneously entered into follow-up after 23 weeks due a false negative serum HBV DNA test (subsequently confirmed by retesting on the COBAS platform). After 10 weeks off-treatment, during which this patient’s infection rebounded, this patient was permitted to undergo a second course of REP 2055 therapy (Table 2).

**Fig 3:**
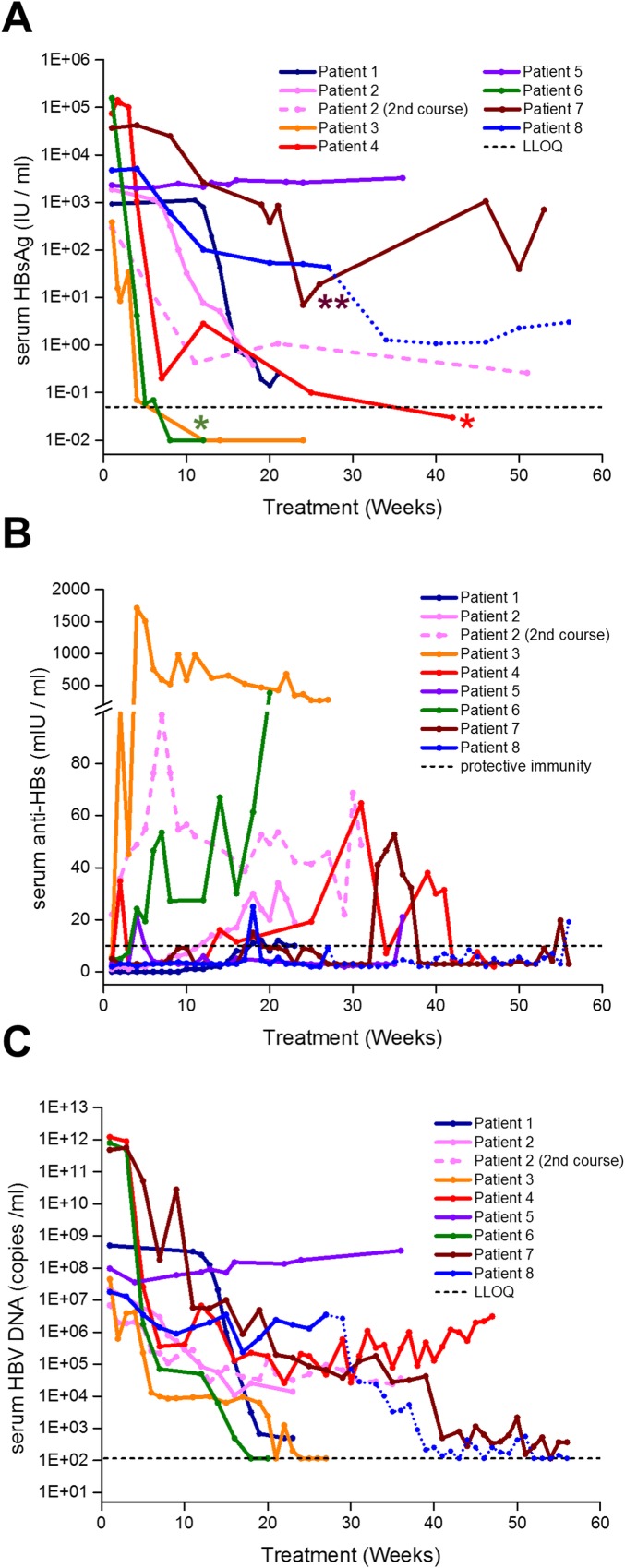
On-treatment antiviral response to REP 2055 monotherapy in patients admitted to the REP 101 protocol. Individual patient, colour coded antiviral responses to NAP monotherapy are shown for serum HBsAg (A), serum anti-HBs (B) and serum HBV DNA (C). LLOQ = lower limit of quantitation. Protective immunity = 10mIU / ml (as defined by the Architect® Assay). *serum HBsAg clearance in these patients until the end of REP 2055 treatment was established via the onsite qualitative HBsAg assay (see methods). ** = transient depression in serum HBsAg associated with increased frequency REP 2055 dosing. Dotted portion of lines for patient 8 in A, B and C indicate an additional 28 weeks of combination therapy with REP 2055 and ETV (see Results and Table 2).

Patients 2 (2^nd^ course), 3, 4, 5 and 6 received multiple weekly doses of REP 2055 (400mg) during the first 2–8 weeks of treatment until serum HBsAg became undetectable using the locally qualified ELISA and then transitioned to once weekly dosing. Patients 7 and 8 only received once weekly doses of 400mg except patient 7, who received multiple weekly doses from weeks 18–24 (Fig 3A) to assess the antiviral effect of more frequent dosing following accepted dosing regimens previously used in human patients for other drugs in this chemical class targeting the liver [14].

Additionally, patients 2 (2^nd^ course) 4 and 7 were allowed to extend their weekly REP 2055 treatment beyond 40 weeks to increase the chance of achieving control of their infection off treatment because REP 2055 therapy was well tolerated at the end of 40 weeks and a partial control of infection was evident in these patients. REP 2055 monotherapy was discontinued early due to futility in patient 5, who experienced no reduction in serum HBsAg (Fig 3A). Patient 8, who experienced a reduction in HBsAg (~ 2 log) but poor reduction (~1 log from baseline) in serum HBV DNA after 28 weeks of monotherapy, was transitioned to 28 weeks of combined therapy with REP 2055 and ETV (0.5mg PO QD) (dotted lines in Fig 3A and 3C and Table 2).

IV infusions in the first two patients dosed were accompanied by peripheral grade 1 hyperemia resolved by switching to DEHP-free IV tubing in these and all subsequent patients. IV administration of REP 2055 at doses of 400mg or greater (even with DHEP-free tubing) were accompanied by grade 1–2 fever, shivering, chills and headache in all patients which resolved 2–8 hours after the infusion was completed. These administration-related side effects responded well to acetaminophen or oral anti-histamine and were further alleviated or eliminated by increasing the infusion time to > 10 hours in subsequent administrations (S2 Table). Intermittent mild gum bleeding and elevated INR was observed at the 1200mg dose level which resolved after dosing was halted and was not observed in any other patient. Asymptomatic hypocalcemia appeared at the onset of 400mg dosing and self-resolved with continued exposure in the first two patients to receive therapy and may be related to the anti-coagulopathy observed above. All subsequent patients receiving REP 2055 received mineral supplementation (calcium, magnesium and vitamin D3) with treatment and serum mineral levels remained normal in these patients.

REP 2055 treatment was generally well tolerated in all patients with no observable drug-related alterations in liver function (discussed further below), kidney function, or general hematological parameters. ECG parameters (measured every 4 weeks) and blood pressure (measured weekly before and after IV infusions) were not affected and the presence of protein, heme or sugar in the urine (measured weekly by dipstick) was never observed for any patient throughout the course of treatment. The onset of mild fatigue was reported by all patients after the 10th week of treatment but the etiology of this fatigue is unclear as patients were also experiencing an antiviral response to treatment during this period (as discussed below). Reported adverse events during the IV infusion and other adverse events considered drug-related in the REP 101 study are listed in S2 and S3 Tables.

**Fig 4:**
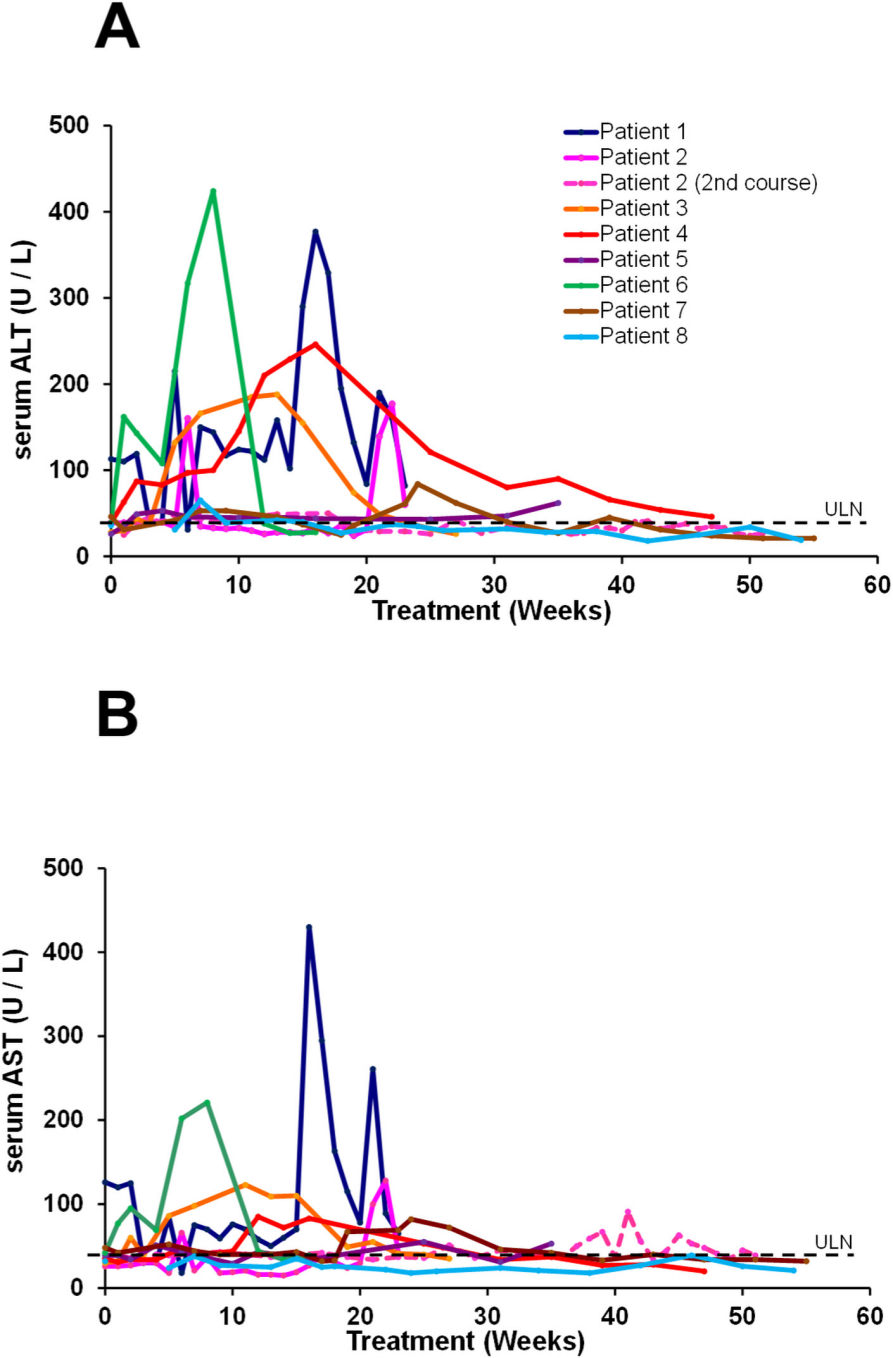
On-treatment serum ALT (A) and AST (B) observations in all patients treated with REP 2055 in the REP 101 study. Individual patient, colour coded test results are provided.

A brief concomitant exposure with low dose peg-IFN (18 and 45 μg over two weeks) was attempted in patient 2 after 36 weeks of REP 2055 monotherapy. Transient anemia and thrombocytopenia (not shown) and mild AST elevations (see Fig 4) developed but reversed with continued exposure to REP 2055. In patient 8, combined exposure to REP 2055 and ETV was well tolerated.

#### Effect on serum HBsAg

Seven of eight patients experienced reductions in serum HBsAg varying from 2.03 to 7.2 log from pre-treatment baseline (Fig 3A, Table 4). Patients who received periods of multiple weekly REP 2055 dosing, (2 (2^nd^ course), 3, 4 and 6) experienced the most rapid reductions in serum HBsAg (Fig 3A). Multiple weekly dosing in patient 7 from weeks 18–24 resulted in significant but transient additional reduction in serum HBsAg that returned to levels maintained previously with once weekly dosing (Fig 3A). Serum HBsAg loss (< 0.05 IU / ml) occurred during monotherapy in patients 3, 4 and 6 (Fig 3A, Table 4). An additional ~1 log reduction in HBsAg was observed during concomitant therapy with ETV in patient 8 (Fig 3A, dotted line).

**Table 4:** Summary of antiviral effect of REP 2055 during treatment and follow-up in the REP 101 study.

Patient	Serum antiviral effect during treatment							Response off treatment
	Maximum log reduction in serum HBsAg observed(baseline [IU / ml]	EOT serum HBsAg (IU / ml)	Maximum anti-HBs detected (mIU / ml)	EOT HBeAg status		EOT anti-HBe status	log reduction in serum viremia relative to pre-treatment (EOT titer [copies / ml])	
1	3.49 (934)	0.30	12	negative ^a^		positive	3.6 (< 500)^c^	NR for 294 weeks ^d^
2	3.67 (1885.4)	0.40	34	negative		positive	3.00 (1.4x10^4^)	viral rebound (attempted retreatment)
2(2^nd^ course)	2.99 (294.5)	0.30	116	negative	positive		2.14 (5.0x10^4^)	viremia persistent at EOT controlled with ETV
3	4.58 (384.1)	0.01	1712	negative ^a^		positive	5.59 (< 116)^d^	NR for 52 weeks ^f^ followed by viral rebound (controlled with ETV)
4	6.87 (74330)	0.01	64.76	negative ^a^		positive ^a^	5.59 (3.1x10^6^)	viremia persistent at EOT controlled with ETV
5	-0.15 (2320.2)	3276	21.5	negative		positive	-1.04 (1.1x10^9^)	contact with patient lost during follow-up
6	7.20 (158180)	0.01	385.7	ND^b^		positive ^a^	9.83 (< 116)^d^	NR for 231 weeks ^g^
7	2.97 (36996)	39.58	52.78	ND		negative	9.11 (372)	viral rebound (admitted to REP 102 protocol)
8	2.03 (4762.5)	43.70	25	negative ^a^	positive ^a^		1.02 (1.7x10^6^)	viral rebound (controlled with ETV)

EOT = end of treatment, IU = international units, ND = not determined (due to sample exhaustion), ETV = entecavir, NR = no evidence of rebound in serum viremia at each follow-up visit for the number of weeks after end of treatment indicated (status at last follow-up visit is indicated in the notes).

^a^ maintained 1 year after withdrawal from REP 2055 therapy.

^b^ HBeAg negative 1 year after withdrawal from REP 2055 therapy.

^c^ LLOQ Serace RT-PCR.

^d^ LLOQ COBAS RT-PCR.

^e^ HBV DNA target not detected, HBsAg 0.07 IU / ml as determined by Abbott Architect quantitative test.

^f^ HBV DNA < 1000 copies / ml, HBsAg undetectable as determined using the onsite qualitative HBsAg ELISA.

^g^ HBV DNA target not detected, HBsAg < 0.05 IU / ml as determined by Abbott Architect quantitative test.

#### Effect on anti-HBs, HBeAg and anti-HBe

HBsAg reduction was accompanied by the appearance of anti-HBs > 10 mU / ml in all patients (Fig 3B) and the levels observed generally correlated with the speed and magnitude of HBsAg clearance (Fig 3A). However, anti-HBs titers were not consistently maintained during therapy except in patient 3. The reduction of serum HBeAg or HBeAg seroconversion was observed in all 7 responder patients except patient 7 (Table 4).

#### Effect on serum HBV DNA

In 6 / 7 patients who experienced significant serum HBsAg declines, serum HBV DNA declines were also substantial (3–12 log) and generally correlated with serum HBsAg reduction. Several patients (2, 3, 4 and 6) experienced protracted periods where serum HBsAg was < LLOQ but where serum HBV DNA was ~ 10^5^ copies per ml (cpm) or greater (Fig 3A and 3C). In patients 1, 3, 6 and 7 there was a reduction of serum HBV DNA to < LLOQ during treatment (Fig 3C). Patient 8 experienced no significant HBV DNA reduction during REP 2055 monotherapy but achieved HBV DNA < 116 cpm during combined treatment with ETV (Fig 3C, dotted line).

#### Serum ALT / AST function accompanying antiviral response

Elevations in serum ALT and AST were observed in 4 / 7 patients after reductions in serum HBsAg had been achieved and were either concomitant with or immediately followed reductions in serum HBsAg and the first (rapid) declines in serum HBV DNA (Figs 3C, 4A and 4B). Transaminase elevations were asymptomatic and self-resolved on treatment within 4–15 weeks after their onset and were judged to be not drug related.

#### Off-treatment outcomes

Patients 1, 3 and 6 all met the criteria for early withdrawal from treatment and received only 23, 27 and 20 weeks of treatment respectively. Evidence of viral rebound (HBV DNA > 1000 copies / ml and HBsAg > 1 IU / ml) was not observed during any follow-up visit for 52 weeks (Table 4). At the time of manuscript submission, no rebound of viremia has been observed in patients 1 and 6 for 270 and 231 weeks after end of treatment respectively (Table 4). Serum viremia was controlled in patient 3 at the 52 week follow-up assessment but had rebounded to pre-treatment levels at the 78 week follow-up visit (data not shown) and serum viremia in this patient was subsequently controlled with ETV. Rebound in serum HBV DNA was accompanied by the re-appearance of serum HBsAg and reduction of serum anti-HBs to < 10 mIU / ml. Patient 7 was permitted to enter the REP 102 study as REP 102 patient 9 without exposure to ETV or TDF (Tables 4 and 5). HBeAg clearance and / or seroconversion was stable 1 year after treatment in patients 1, 2, 3, 6 and 8 (Table 4).

**Table 5:** Summary of antiviral effect of REP 2139-Ca and immunotherapy during treatment and follow-up in the REP 102 study.

Patient	Antiviral effect during treatment						Response off treatment
	Log reduction in serum HBsAg observed at end of REP 2139-Ca monotherapy (baseline IU / ml)	Log reduction in serum HBsAg observed at EOT (IU / ml)	maximum anti-HBs detected (mIU / ml)	EOT HBeAg status	EOT anti-HBe status	log reduction in serum viremia relative to pre-treatment (EOT titer [copies / ml])	
1	1.14 (168950)	NE	1.22	positive	negative	0.42 (3.75x10^8^)	viremia persistent at EOT controlled with ETV
2	5.57 (70050)	6.37 (0.03)	708.37	negative	positive	5.71 (1.21x10^3^)	NR for 112 weeks (rebound controlled by ETV)
3	6.13 (13400)	6.13 (0.01)	1250	negative^a^	positive ^a^	4.86 (2.3x10^3^)	NR for 135 weeks ^e^
4	4.01 (3450)	5.06 (0.03)	795.47	positive ^b^	positive	5.11 (2.01x10^3^)	NR for 123 weeks ^f^
5	-0.02 (7676.5)	NE	0.79	positive	negative	0.76 (7.93x10^6^)	viremia persistent at EOT controlled with ETV
6	2.45 (50994.9)	6.23 (0.03)	381.57	negative ^a^	positive ^a^	4.83 (2.55x10^3^)	NR for 17 weeks (rebound controlled by ETV)
7	3.44 (87690.1)	6.94 (0.01)	156.32	negative ^a^	positive ^a^	5.91 (1.23x10^3^)	NR for 12 weeks (rebound controlled by ETV)
8	3.87 (72968)	6.56 (0.02)	512.34	negative ^a^	positive ^a^	6.26 (541)	NR for 32 weeks (rebound controlled by ETV)
9	2.79 (17989)	1.96 (198.75)	258.53	positive	negative	2.39 (2.86x10^3^)	rebound (controlled by ETV)
10	0.00 (123980)	NE	4.15	positive	negative	0 (> 9.89x10^8^)	viremia persistent at EOT controlled with TDF
11	7.1 (> 125000)	6.80 (0.02)	195.08	negative ^a^	positive ^a^	6.93 (< 116 ^d^)	NR for 137 weeks ^g^
12	4.88 (1504.11)	4.88 (0.02)	462.22	positive	negative ^c^	3.46 (1.8x10^3^)	NR for 37 weeks (rebound controlled by ETV)

EOT = end of treatment, IU = international units, NE = no exposure to immunotherapy (non-responder), ETV = entecavir, TDF = tenofovir disoproxil fumarate, NR = no evidence of rebound in serum viremia (serum HBV DNA < 1000 copies / ml, serum HBsAg < 1 IU / ml) at each follow-up visit for the number of weeks after the end of treatment indicated (status at last follow-up visit is indicated in the notes).

^a^ maintained 1 year after withdrawal from REP 2139-Ca therapy.

^b^ HBeAg continually dropped during treatment and at the end of treatment was close to the cut-off threshold for HBeAg negativity.

^c^ reported value for anti-HBe was close to the cut-off threshold for anti-HBe positivity.

^d^ LLOQ Roche cobas PCR.

^e^ HBV DNA = 186 copies / ml, HBsAg = 0.89 IU / ml.

^f^ at 132 weeks follow-up, HBV DNA = 3360 cpm and HBsAg = 1.62 IU / ml.

^g^ HBV DNA target not detected, HBsAg < 0.05 IU / ml.

HBV DNA either present at treatment withdrawal (patient 2 and 4) or that rebounded during follow-up after treatment withdrawal (patient 3 and 8), was controlled by rescue with ETV. Transition to ETV was not accompanied by any adverse events in any patient. Elevated ALT / AST levels were not observed during follow-up or viral rebound in any patient (data not shown). Fibroscan analysis was performed in patients 1, 2 and 3 during follow-up but no conclusive changes in liver fibrosis status relative to baseline at the start of treatment were evident.

### REP 102 protocol (REP 2139-Ca + immunotherapy)

#### Dosing, safety and tolerability

**Fig 5:**
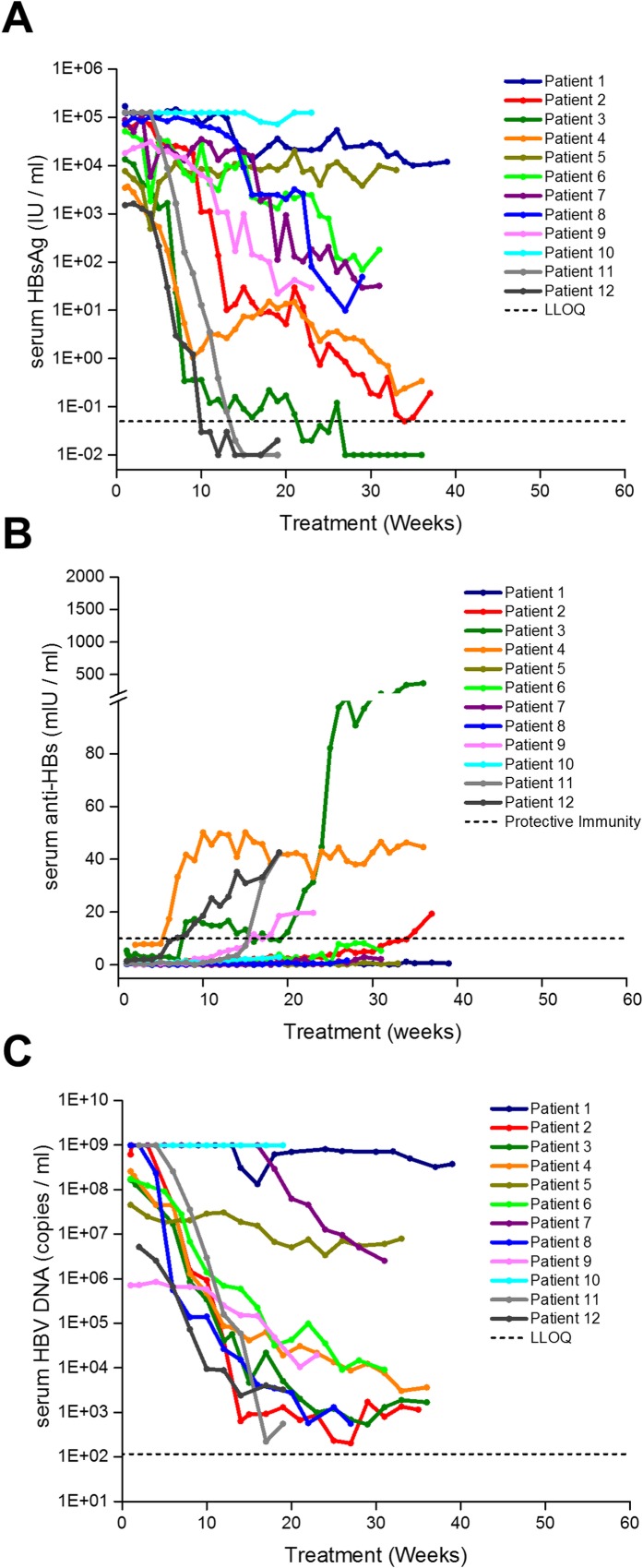
On-treatment antiviral response to REP 2139-Ca monotherapy therapy in patients admitted to the REP 102 protocol. Individual patient, colour coded antiviral responses to NAP monotherapy are shown for serum HBsAg (A), serum anti-HBs (B) and serum HBV DNA (C). LLOQ = lower limit of quantitation. Protective immunity = 10mIU / ml (as defined by the Architect® Assay).

REP 2139-Ca was given as a weekly 500mg IV infusion (to account for the increased molecular weight of REP 2139 vs REP 2055) with mineral supplementation as described above in the REP 101 protocol. Following an interim safety and efficacy analysis of all REP 102 study patients after completion of at least 20 weeks of REP 2139-Ca monotherapy (Fig 5), patients with no significant adverse events with > 2 log reduction from baseline in serum HBsAg and serum HBV DNA during monotherapy (9 of 12 patients) were permitted to transition to combination treatment with short term immunotherapy while continuing REP 2139-Ca treatment.

**Fig 6:**
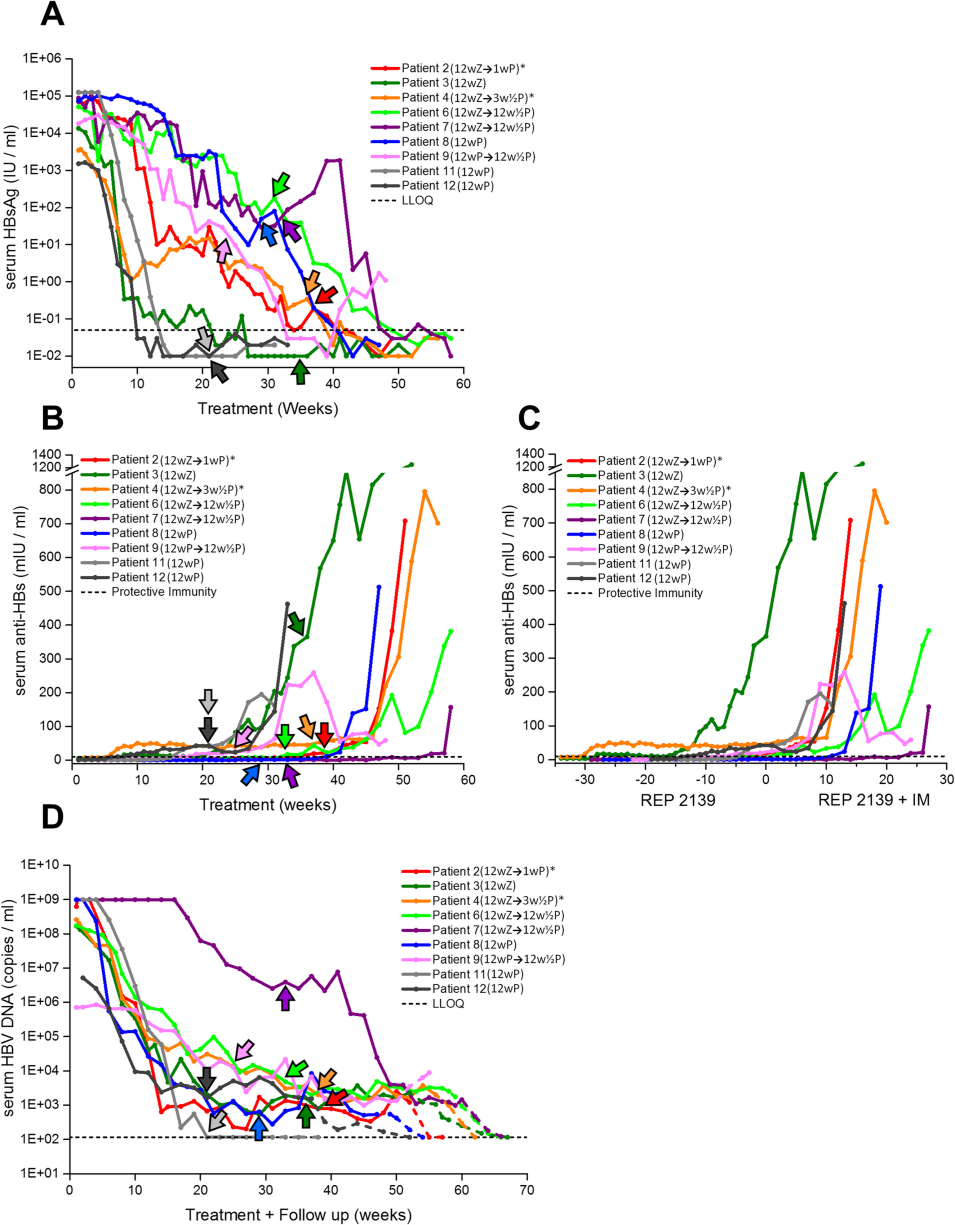
On-treatment antiviral response in the nine REP 2139-Ca patients in the REP 102 protocol who responded to monotherapy and proceeded to combination therapy with REP 2139-Ca and pegylated interferon alpha 2a or thymosin alpha 1. Individual patient, colour coded antiviral responses to total therapy are shown for serum HBsAg (A), serum anti-HBs (B), serum anti-HBs normalized to the start of combination therapy (C) and serum HBV DNA (D). In A, B and D the colour coded arrows indicate the start of immunotherapy for each respective patient. In (C), negative numbers indicate REP 2139-Ca monotherapy and positive numbers from 0 onward indicate combination therapy. Immunotherapy exposure for each patient (see methods) are indicated in the figure legend as follows: Z = thymosin alpha 1, P = pegylated interferon alpha 2a, ½ P = extended pegylated interferon exposure at ½ dose (with REP 2139-Ca at ½ dose). Dotted coloured lines in (D) indicate initial serum HBV DNA responses off-treatment.* = patients with inadvertent exposure to 1–3 weeks of peg-IFN at the end of treatment.

The timing of the transition from REP 2139-Ca monotherapy to combination therapy with REP 2139-Ca and immunotherapy is indicated for each patient in Fig 6 (see arrows and legend). Immunotherapies used were thymosin alpha 1 (thymosin; as Zadaxin®) starting at ½ dose the first week (1.6mg) followed by twice weekly 1.6mg doses via subcutaneous injection for 11 additional weeks or pegylated interferon alpha 2a (peg-IFN; as Pegasys®) via weekly subcutaneous injection with dose escalation over the first 3 weeks (with 18, 38 and 90ug weekly doses) followed by an additional 9 weeks at 180 ug / week.

In three patients with weaker anti-HBs responses following initial immunotherapy, extended immunotherapy was permitted. Patients 6 and 7, who initially received thymosin received peg-IFN for 12 weeks at ½ dose (250mg REP 2139-Ca and 90ug Pegasys® respectively). Patient 8 was also permitted to extend combination therapy with peg-IFN for an additional 12 weeks at ½ dose. Due to delays in receiving antiviral test results at the trial site, REP 102 patients 2 and 4 received 1 and 3 weeks of peg-IFN after completing 12 weeks of thymosin exposure after which treatment was halted (due to delays in receiving test results) as these patients had established strong anti-HBs responses with previous thymosin exposure (Fig 6). Patient 9 was permitted to complete a total 48 weeks of peg-IFN treatment after withdrawal of REP 2139-Ca despite evidence of rebound with lowered REP 2139-Ca dosing (data not shown). All patients were compliant with the prescribed dosing regimens except for patient 7, who had REP 2139-Ca dosing withheld (as a precautionary measure) while continuing immunotherapy during treatment weeks 36–38 during the resolution of an unrelated infectious gastroenteritis.

Unlike REP 2055, REP 2139-Ca IV infusion was not accompanied by the development of significant fever, shivering or headache and infusion times were gradually reduced to 2 hours with only the occasional occurrence of mild fever, shivering or itching which rapidly self-resolved in the absence of any supportive therapy. Serum mineral levels were normal in all patients and REP 2139-Ca treatment (monotherapy and when combined with immunotherapy) was generally well tolerated in all patients with no observable drug-related alterations in liver function (discussed further below), kidney function, or general hematological parameters. ECG parameters (measured every 4 weeks) and blood pressure (measured weekly) were not affected and the presence of protein, heme or sugar in the urine (measured weekly by dipstick) was never observed for any patient throughout the course of treatment. The onset of mild fatigue was reported by all patients similar to REP 2055 monotherapy. Symptoms most frequent with REP 2139-Ca treatment included dyspepsia, reduced appetite and fever and mild to moderate dysphagia and or hair loss that developed in all patients receiving REP 2139-Ca by the end of their combination exposure to immunotherapy or during initial follow-up after treatment was halted. Reported IV infusion events and other adverse events considered treatment-related in the REP 102 study are listed in S4 and S5 Tables.

Before the end of REP 2139-Ca combination therapy with peg-IFN, patient 12 had developed thrombocytopenia (20 x 10^9^ platelets / L) which was accompanied by petechial rash over the entire body. These symptoms and signs were attributed to Pegasys® exposure in this patient. After withdrawal from treatment and admission to hospital for supportive steroid therapy, the platelet count in this patient recovered to normal levels within two weeks. The patient was released from hospital and entered into the regular follow-up schedule.

#### Effect on serum HBsAg

Nine of twelve patients on REP 2139-Ca monotherapy experienced reductions in serum HBsAg varying from 2.79 to 7.10 log from pre-treatment baseline (Fig 5A, Table 5) and three of these patients (3, 11 and 12) experienced HBsAg loss during monotherapy. These 9 patients continued on to combination treatment with immunotherapy during which HBsAg loss was maintained in patients 3, 11 and 12 and serum HBsAg continued to decline in the other 6 patients who eventually achieved HBsAg loss by the end of therapy (Fig 6A, Table 5).

#### Effect on anti-HBs, HBeAg and anti-HBe

The appearance of anti-HBs > 10 mIU / ml during REP 2139-Ca monotherapy was observed in 5 patients during REP 2139-Ca monotherapy (Fig 5B) which was also correlated with the extent of serum HBsAg reduction. Serum titers that became detectable above 10 mIU / ml appeared more stable than in REP 2055 treated patients.

In the 9 REP 102 patients who received add-on immunotherapy, rapid and substantial increases in anti-HBs titres were observed in all patients receiving either thymosin or peg-IFN (Fig 6B). These increases in serum anti-HBs appeared to be generally correlated with the addition of immunotherapy (Fig 6C) but also were more rapid in patients with < 10 IU /ml of HBsAg at the start of immunotherapy (Fig 6A and 6C). The rebound in serum HBsAg and loss of anti-HBs in patient 9 (Fig 6A, 6B and 6C) was correlated with the transition to ½ dosing of REP 2139-Ca and peg-IFN in the extended course of immunotherapy this patient received.

The loss of serum HBeAg and HBeAg seroconversion was observed in all 9 patients responding to REP 2139-Ca monotherapy except patient 9 (Table 5).

#### Effect on serum HBV DNA

In the 9 / 12 patients who experienced significant serum HBsAg declines, serum HBV DNA declines were also substantial (3–12 log) and the start of these declines were generally correlated with the onset of detectable serum HBsAg reductions (Fig 5C). Three patients (3, 7 and 12) experienced protracted periods during monotherapy where serum HBsAg was < LLOQ but where serum HBV DNA was ~ 10^4^ copies per ml (cpm) or greater (Fig 5A and 5C).

With the addition of immunotherapy, additional clearance of serum HBV DNA was observed in patient 7 and in all other patients HBV DNA reached a plateau of 3000–5000 cpm which was stable throughout combination therapy (Fig 6D).

#### Serum ALT / AST function accompanying antiviral response

**Fig 7:**
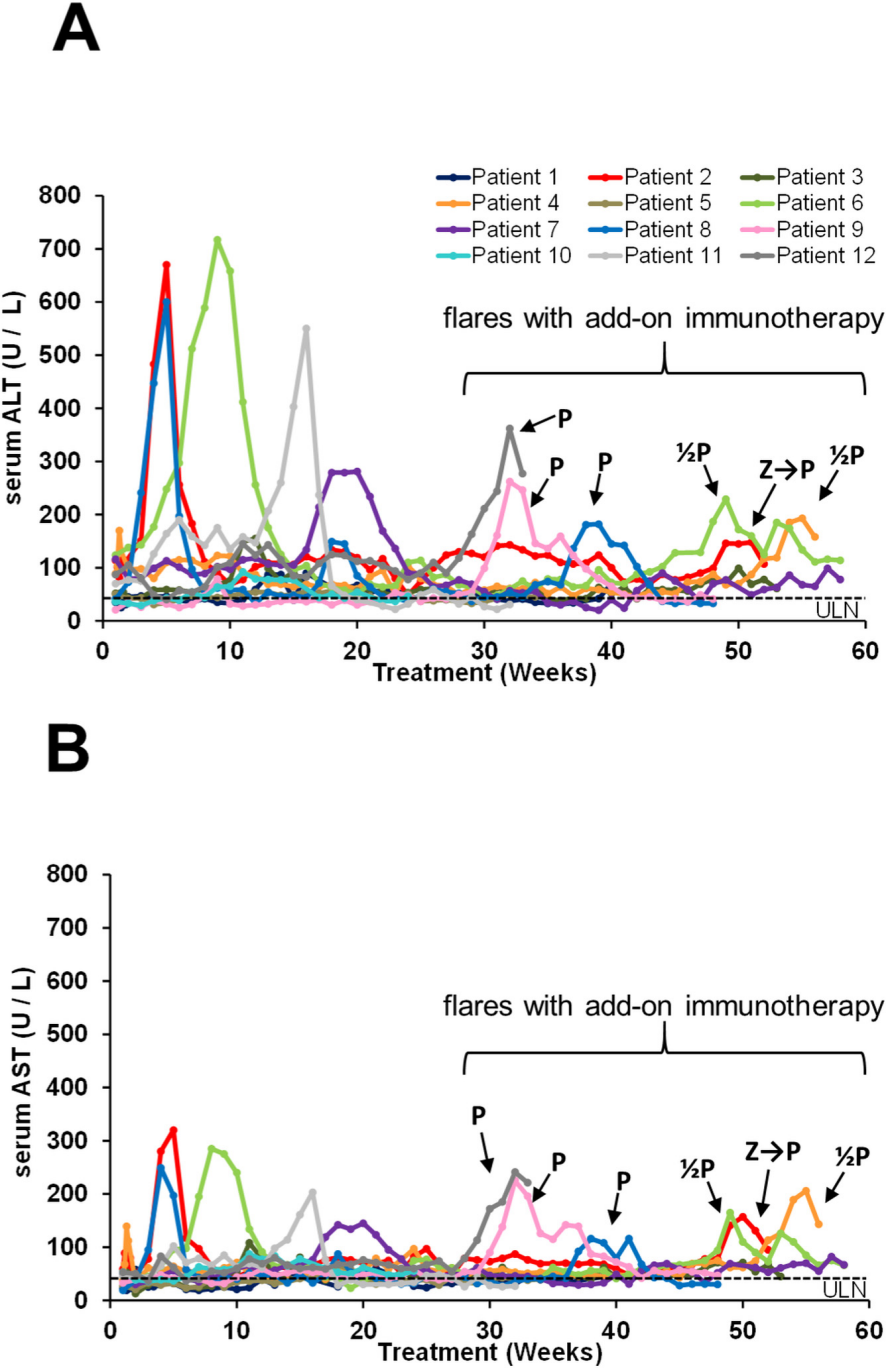
On-treatment serum ALT (A) and AST (B) observations in all patients treated with REP 2139-Ca and immunotherapy in the REP 102 study. Individual patient, colour coded test results are provided. The immunotherapy present at the time of ALT flare is indicated with arrows: Z = thymosin alpha 1, P = pegylated interferon alpha 2a, ½ P = pegylated interferon exposure at ½ dose (with REP 2139-Ca at ½ dose).

During REP 2139-Ca monotherapy, elevations in serum ALT and AST were observed in 5 /9 patients after reductions in serum HBsAg had been achieved except in patient 8 (Fig 7A and 7B) and were either concomitant with or immediately followed the first rapid declines in serum HBV DNA. These ALT / AST elevations were asymptomatic and self-resolved with continuing treatment within 4–15 weeks after their onset and were judged to be not drug related. New or additional ALT / AST elevations in patients 2, 4, 8, 9 and 12 were observed after the addition of immunotherapy which appeared to be restricted to peg-IFN exposure (Fig 7A and 7B). These elevations were generally smaller than those observed during REP 2139-Ca monotherapy, were self-resolving, otherwise asymptomatic and did not appear to be associated with any additional productive antiviral effect on serum viremia.

#### Off-treatment outcomes

Of the nine patients in the REP 102 study who received REP 2139-Ca combined with immunotherapy, eight continued to experience serum HBV DNA reductions off treatment and reached serum HBV DNA < LLOQ during the initial period of follow-up (Fig 6D, dotted lines). In these eight patients viral rebound (HBV DNA > 1000 copies / ml, or HBsAg > 1 IU/ ml) was subsequently observed in two patients at ~ 3 months (patients 6 and 7), in two patients at ~ 6 months (patients 8 and 12), in one patient at ~ 14 months (patient 2) and one patient at 123 weeks (Table 5). Before rebound occurred in these patients, suppression of HBV DNA and HBsAg was persistent at each visit. In two patients (3 and 12) no evidence of viral rebound has been observed off treatment 132–137 weeks after stopping all treatment (Table 5). HBeAg seroconversion was maintained for 1 year after follow-up in patients 3, 6, 7, 8 and 11 (Table 5). Rebound in serum HBV DNA during follow-up was well controlled in all cases by rescue with ETV. In the three non-responder patients, viremia persisting at the end of treatment was well controlled with ETV or TDF. Transition to ETV or TDF was not accompanied by any adverse events. Rebound in HBV DNA in all patients was also accompanied by re-appearance of serum HBsAg and reduction of serum anti-HBs to < 10 mIU / ml. Elevated ALT / AST levels were not observed during follow-up, even during viral rebound, in any patient. Fibroscan analysis of patients conducted after 2 years of follow-up (at this time 9/12 patients were receiving ETV) showed no change in liver fibrosis relative to pre-treatment status (data not shown).

## Discussion

### NAP tolerability

NAPs are phosphorothioated oligonucleotides (PS-ONs) whose antiviral effect against HBV infection is independent of nucleotide sequence [11, 12]. Unlike standard antisense compounds, NAPs can be more easily engineered to remove the secondary pro-inflammatory / immunostimulatory effects of single stranded nucleic acids while maintaining their antiviral activity *in vivo* [13, 15]. The primary tolerability issue with REP 2055 in the REP 101 study was IV administration related side effects similar to those reported for other PS-ONs administered to humans by IV infusion [16, 17] which was well controlled with REP 2139-Ca in the REP 102 study. Additionally, short term combination therapy with REP 2139-Ca and thymosin or peg-IFN up to 26 weeks was well tolerated. The liver flares during monotherapy with either REP 2055 or REP 2139-Ca occurred following initial reductions of serum HBsAg and HBV DNA, were self-resolving with continued NAP therapy and were not accompanied by any symptoms or serological evidence of liver dysfunction. These liver flares are also routinely observed with treatment with interferons, and are thought to be evidence of re-activation of immune function in the liver [18]. These liver flares may be evidence of reactivation of the immune response in the liver with reduction of circulating HBsAg (discussed below) but additional analysis of T-cell response during transaminase flares concomitant with serum HBsAg reduction in future trials will be required to address this hypothesis.

PS-ONs increase mineral elimination in the urine [19] and the resulting compensatory response includes liberation of mineral stores (along with heavy metals if present) from the bones into the circulation. All study patients and untreated control subjects at the trial site were shown to have substantial pre-existing heavy metal loads (data not shown) as a result of chronic heavy metal exposure known to be endemic at the trial site [20–22]. The 2’ ribose modifications present in REP 2139 are absent in REP 2055 (Fig 2) and serve to reduce immunoreactivity [23, 24] but also block degradation by endonucleases [25], rendering REP 2139 substantially more stable than REP 2055 and leading to greater accumulation of REP 2139-Ca in pre-clinical models with chronic exposure (A. Vaillant, unpublished data). Thus chronic exposure to REP 2139-Ca likely establishes a greater mineral elimination (and heavy metal liberation) than REP 2055, consistent with development of hair loss, dysphagia and dysgeusia observed in patients with REP 2139-Ca therapy but not with REP 2055.

These symptoms have not been previously reported with the clinical use of PS-ONs, however, these studies are the first to be conducted in a locale where substantial heavy metal exposure is highly endemic in the population. Importantly, in a currently ongoing clinical trial with REP 2139-Ca in Caucasian patients (REP 301 study, NCT02233075) in a European locale where patients with heavy metal exposure were excluded from participation, none of these symptoms have been observed with comparable REP 2139-Ca exposure in combination with peg-IFN (A. Vaillant, unpublished data).

These clinical observations underscore the importance of being aware of the potential complications of the enhanced mineral elimination known to occur with PS-ON therapy in general and further suggest that mineral supplementation should accompany any oligonucleotide based therapy, regardless of the disease state in patients receiving therapy.

### Therapeutic implications of NAPs

Although further controlled trials will be required to confirm the initial safety observations and findings of antiviral activity observed in the proof-of-concept studies presented here, NAP monotherapy (either REP 2055 or REP 2139-Ca) was clearly accompanied by substantial reductions or clearance of serum HBsAg and appearance of anti-HBs < 10 mIU / ml with concomitant reductions in serum HBV DNA in 16 of 20 patients with HBeAg+ chronic HBV infection. These effects are very similar to the antiviral effects of REP 2055 reported in Pekin ducks with previously established persistent DHBV infection [13]. No evidence of virologic breakthrough was observed during NAP monotherapy lasting as long as 54 weeks or during combined treatment with immunotherapy in these initial studies. Moreover, two patients receiving an initial course of NAP therapy (REP 2055 in patients 2 and 7 in the REP 101 protocol), who experienced viral rebound during follow-up off-treatment responded identically with a second course of NAP therapy (REP 101 patient 2 responded to a second course of REP 2055 following viral rebound and in REP 101 patient 7, who became REP 102 patient 9, viral rebound after withdrawal of REP 2055 responded to REP 2139-Ca) suggesting the development of resistance to NAP therapy may be unlikely, however larger clinical trials will be required to ascertain if viral resistance to NAP therapy can occur.

The absence of rebound of HBV infection after cessation of REP 2055 treatment in 3/7 patients for 52 weeks parallels the sustained control of DHBV infection observed *in vivo* after REP 2055 monotherapy in ducks, which was correlated with transcriptional inactivation and clearance of cccDNA [13]. The more durable and complete suppression of serum viremia achieved in 2 patients who received REP 2055 monotherapy (where serum HBV DNA is not detectable and HBsAg is not currently detectable or detectable at residual levels, see Table 4) may reflect the establishment of control of cccDNA and / or integrated HBV genomes in these patients but this remains to be proven. More frequent dosing with REP 2055 appeared to improve the clearance of HBsAg and allow the achievement of serum HBsAg < LLOQ however similar reductions in serum HBsAg were achieved with REP 2139-Ca in the absence of periods of more frequent dosing which may be related to the increased stability of REP 2139.

When REP 2055 was used therapeutically *in vivo* against persistent DHBV infection, many animals maintained substantial titers of serum DHBV DNA with no detectable levels of serum DHBsAg, moreover at the end of REP 2055 treatment in many ducks, DHBsAg was readily detectable in the liver but not detectable in the serum, suggesting that NAPs selectively block the release of DHBV subviral particles from infected hepatocytes by REP 2055 [13]. The persistence of significant serum HBV DNA titers while serum HBsAg was maintained near or < LLOQ with REP 2055 or REP 2139-Ca monotherapy in human patients has striking similarity to these effects observed with REP 2055 in ducks. Given that subviral particles constitute the bulk of serum (D)HBsAg in both DHBV infected ducks and human HBV infection [2, 26], these correlative observations of the effects of NAP monotherapy in DHBV and HBV infection suggest that selective blockage of subviral particle secretion by NAPs may also be occurring in human patients.

The continued suppression of viremia after withdrawal of NAP therapy may be related to reduction or clearance of serum HBsAg during therapy. Serum HBsAg sequesters anti-HBs but importantly has also been shown to have direct immunoinhibitory properties *in vitro* and *in vivo* [27–32]. HBsAg is the most abundantly circulating viral antigen and it may exert direct inhibition of the immune response (both adaptive and innate) to HBV infection. Therefore, when HBsAg is removed or substantially lowered, some form of restoration of the host immune response may occur, allowing for a functional control of infection to be established which persists after NAP therapy is withdrawn.

REP 2139-Ca was safely combined with short term duration (12–26 weeks) of either thymosin or peg-IFN, which resulted in a rapid and robust increase in anti-HBs production in all patients. More importantly, even with short term exposure to these immunotherapies, serum viremia did not rebound after withdrawal of treatment in 8/9 patients and although variable in duration, remained suppressed for extended periods (> 2 years) in 3 / 9 patients, suggesting that restoration of immune control of infection with immunotherapy might be easier to achieve with this novel combination therapy approach. These effects suggest a potentially synergistic improvement in the antiviral effect of peg-IFN and thymosin when combined with REP 2139-Ca. This synergism may be related to the reduction or absence of serum HBsAg in these patients as sustained control of infection after peg-IFN monotherapy is highly correlated with HBsAg clearance [3, 4]. The HBsAg protein itself has been shown to have direct immunoinhibitory properties on both innate and adaptive immune function [27–32] which may also interfere with the downstream effects of immunotherapeutic agents.

The persistence of serum HBV DNA in the absence of detectable serum HBsAg and in the presence of anti-HBs > 10 mIU / ml is a highly unusual clinical observation that was nevertheless common with NAP treatment in both REP 101 and 102 protocols and raises some important theoretical considerations: the amount of HBsAg present in the titers of HBV DNA persisting when serum HBsAg is < LLOQ may be below the LLOQ of the HBsAg test or may indicate the presence of immune escape HBV variants, which have been previously documented in human HBV infection [33–36]. Evaluation of the population genotypes of the HBV present in these patients during NAP therapy by deep sequencing is currently underway and may help to answer some of these questions. Fluctuations in anti-HBs levels were observed in the REP 101 study during treatment while HBsAg and HBV DNA declines were stable. However, fluctuations in serum anti-HBs were not observed in the REP 102 study during monotherapy. REP 2139 is significantly more stable than REP 2055 in human plasma (as discussed above) and may provide a more uniform antiviral effect between weekly doses than REP 2055. This reduced stability of REP 2055 could be an underlying factor in the fluctuating antibody response observed in the REP 101 study.

### Future prospects

Both the REP 101 and 102 trials presented in this article and a third trial (REP 201 –NCT02726789) have been previously disclosed [37]. The REP 201 trial was a very small exploratory trial where REP 2139-Ca treatment with a full course of peg-IFN was attempted in three treatment naïve patients and two patients concurrently receiving ETV. Although similar responses in serum HBsAg, anti-HBs and HBV DNA were observed during treatment as in the REP 102 protocol, the very small patient numbers employed prevent conclusions on improvements in antiviral performance relative to those seen in the REP 102 trial. However the REP 201 trial suggests that NAPs might be used safely in combination with peg-IFN and ETV or other nucleos(t)ide based HBV polymerase inhibitors. This triple combination approach is currently being evaluated in patients with HBeAg negative chronic HBV infection in the REP 401 protocol (NCT02565719, see below).

The results of the REP 101 and REP 102 studies describe the preliminary steps of the development of NAPs for treatment of chronic HBV infection. They indicate that continued clinical evaluation of the antiviral efficacy of NAP-based combination therapy in chronic HBV infection is warranted. Although control groups were not possible in these studies, effective clearance of HBsAg and HBV DNA and the appearance of anti-HBs is unusual. Of greater potential interest is the potential synergistic improvement in the antiviral effects of immunotherapeutic agents when combined with NAP therapy as these might potentially be related to HBsAg reduction. Even with a limited course of immunotherapy, 8/9 patients were able to maintain suppression of serum viremia off-treatment, with many of these maintaining suppression longer than 1 year.

The proportion of patients able to maintain suppression of viremia off-treatment and the duration of this suppression may be significantly improved if NAP therapy was combined with a full course of immunotherapy (i.e. 48 weeks) or if used in combination therapy in patients already seroconverted for HBeAg or currently receiving therapy with ETV or TDF, agents which have been shown to lower intrahepatic cccDNA levels [38, 39]. These concepts are currently being evaluated in the protocol REP 401 protocol (NCT02565719), a randomized, controlled trial assessing the safety and efficacy of triple combination antiviral therapy with NAPs, peg-IFN and TDF in Caucasian patients with HBeAg negative chronic HBV.

Finally, additional trials with REP 2139-Ca should be conducted in patients from diverse ethnic groups and harbouring different HBV genotypes to investigate if the antiviral responses with NAP therapy observed in the patients in these studies could also be achieved in patients from other geographic regions.

## Supporting Information

S1 File Trend Statement Checklist. (PDF)

S2 File REP 101 study protocol and amendment. (PDF)

S3 File REP 102 study protocol and amendment. (PDF)

S1 Table Ishak and Knodell scores from pre-treatment liver biopsies in REP 101 patients. (DOCX)

S2 Table IV infusion adverse reactions in the REP 101 study. (DOCX)

S3 Table Treatment related adverse events in the REP 101 study. (DOCX)

S4 Table IV infusion adverse reactions in the REP 102 study. (DOCX)

S5 Table Treatment related adverse events in the REP 102 study. (DOCX)
