# Supplementary material for: Safety and Efficacy of Nucleic Acid Polymers in Monotherapy and Combined with Immunotherapy in Treatment-Naive Bangladeshi Patients with HBeAg+ Chronic Hepatitis B Infection
Source: PLoS One. 2016 Jun 3;11(6):e0156667. doi: 10.1371/journal.pone.0156667 (PMC4892580; doi:10.1371/journal.pone.0156667)
Supplement: S2 File — (PDF) [file pone.0156667.s002.pdf]

Clinical Trial Protocol

STUDY TITLE: Therapeutic safety and efficacy of REP 9AC in HBV or HCV infected patients.

PROTOCOL IDENTIFICATION NUMBER: REP101

STUDY SPONSOR: REPLICor Inc., Laval, Canada

INSTITUTION: Bangabandhu Sheikh Mujib Medical Center, Dhaka, Bangladesh

STUDY STATUS: IRB Approved/

STUDY DESIGN: Treatment, open label, safety / efficacy study, adaptive trial

ESTIMATED ENROLLMENT: 12 to 24 patients

ESTIMATED STUDY START DATE: January 2009

STUDY AUTHORS: Mamun-Al-Mahtab, MD (Bangladesh), Principal Investigator
Andrew Vaillant, Ph.D. (REPLICor Inc.)
Michel Bazinet, MD (REPLICor Inc.)

1. INTRODUCTION

Chronic hepatitis B and C are long term conditions caused by infection of the body with the hepatitis B (HBV) and C (HCV) viruses. These infections often result in inflammation or scarring of the liver and can eventually lead to liver cirrhosis and liver failure. These infections are also one of the major cause of the development of hepatocellular carcinoma (liver cancer).

Hepatitis B. Although some drugs have been approved to treat chronic hepatitis B infections, they do not provide a complete cure except in rare cases (a cure generally means that a person loses the hepatitis B virus and develops protective surface antibodies). However, these drugs significantly decrease the risk of liver damage from the infection by slowing or stopping the virus from reproducing. Amongst the problems associated with currently available drugs are the tendency to develop resistance and the lack of clearance of the virus from the hepatocytes. There is clearly a need to identify new drugs that can benefit patients with chronic hepatitis B infections. REP 9AC is a 40mer phosphorothioate oligonucleotide that has been shown to have low toxicity and to be highly effective to treat hepatitis B infection in animals. It has been shown to be effective in protecting animal from infection and to treat animals already infected.

We have evaluated the efficacy of REP 9AC in duck hepatitis B in collaboration with Dr. Allison Jilbert (University of Adelaide, Australia), who is a recognized expert on HBV infections. Our results in ducks suggest that our drug can completely eliminate the disease in a large proportion (50%) of animals following only 4 weeks of treatment. In other words, following cessation of the treatment, the viral titer does not return. If these results are replicated in humans, this will result in a paradigm shift in how patients with hepatitis B are treated. Currently available drugs can only control the disease. Once they are stopped, the viral titer returns.

This proposed study is designed to demonstrate that REP9AC can be well tolerated when given to human patients chronically infected with HBV and to evaluate if a reduction of viral titers can be observed when REP 9AC is administered as a monotherapy.

Hepatitis C. Recent advances in the treatment of hepatitis C, using interferon and ribavirin combination therapy, have brought overall response rates to about 50% but these responses are associated with significant costs and toxicities. In view of the lack of response of many patients to standard therapy and in view of its associated toxicities, the development of new therapeutic strategies is critically important. Amphipathic polymers have been shown to have broad spectrum antiviral activities and to have very favorable pharmacokinetics in the liver making them ideal candidates for the therapy of viral hepatitis B and C.

In collaboration with Dr. Jake Liang, (Head, Liver Diseases Branch, National Institute of Health, USA), we have demonstrated that amphipathic DNA polymers are potent HCV entry inhibitors in vitro. In vivo, REP 9AC provides complete protection from HCV infection in naïve animals. REP 9AC is perfectly suited for the treatment of chronic HCV infection as exposure to this drug will allow the liver to naturally eliminate infected cells by halting the continual cycle of infection of healthy liver cells. REP 9AC may eventually replace pegylated interferon and ribavirin as the standard of care owing to its activity against all genotypes of HCV and its novel mechanism of action, which does not promote the generation of drug resistance.

An entry inhibitor could be useful when used in combination therapy or could be effective as a monotherapy. This proposed study is designed to demonstrate that REP 9AC can be well tolerated when given to patients chronically infected with HCV and to evaluate if a reduction of viral titers can be observed when REP 9AC is administered as a monotherapy.

REP 9AC. REPLICor's technology utilizes the novel properties of modified oligonucleotides (phosphorothioate oligonucleotides) as amphipathic polymers to inhibit interactions critical for viral activity. This technology is active in vitro against all known families of enveloped viruses. REPLICor's proof of concept compounds, REP 9 and REP 9C and its lead compound, REP 9AC have also demonstrated potent antiviral activity in vivo against the following viral infections: HCV, HBV (DHBV), Cytomegalovirus, HSV-2, Ebola, influenza and respiratory syncytial virus.

REP 9, REP 9C and REP 9AC have been administered at therapeutically active doses in acute and chronic regimens by multiple routes of administration (parenteral, oral, topical and aerosol) in mice, rats, hamsters, guinea pigs, ducks and non human primate species with no detectable side effects . Moreover, this class of chemical compounds (phosphorothioate oligonucleotides) are known to have been well tolerated in human patients in several clinical trials.

REPLICor has verified the cGMP manufacture of REP 9AC which has been certified for human parenteral administration. The pharmacokinetic behavior of REP 9AC is similar to other compounds from the same chemical class (phosphorothioate oligonucleotides) which achieve long lasting, therapeutic liver concentrations typically using a single 200-400mg dose delivered once every week or once every other week in human patients.

2. OBJECTIVES

Primary objective

> To demonstrate that REP 9AC is well tolerated when given intravenously to patients infected with either chronic hepatitis B or C.

Secondary objective

> To evaluate the viral titer response following the intravenous administration of REP 9AC in patients infected with either chronic hepatitis B or C.

3. STUDY DESIGN AND SCHEDULE OF ASSESSMENTS

Treatment Period: Maximum of 40 weeks

Follow up: Minimum of 24 weeks

Dosing interval: Once every 7 days

Dosing route: IV (120min infusion with drug diluted in 250cc normal saline)

Dose levels: 0, 100, 200, 400 and 800mg (dose escalation)

Number of patients: 12 to 24

Patient inclusion criterion (HBV)

- Age between 18 and 55
- HBsAg+ for at least 6 months prior to initiation of treatment.
- HBeAg+
- HBV titer > 20000 copies/ml at start of treatment

- Treatment naïve
- HIV/HDV/HCV negative
- Compensated liver disease
- Ishak score ≤ 2
- Non cirrhotic
- No known active CMV infection
- Willingness to utilize adequate contraception while being treated with REP 9AC and for 6 months following the end of treatment
- Adequate venous access allowing weekly intravenous therapies and blood tests

Patient inclusion criterion (HCV)

- Age between 18 and 55
- HCV positive for at least 6 months prior to initiation of treatment
- Genotype 3
- HCV titer >3log IU/ml at start of treatment
- Treatment naïve
- HIV/HDV/HBV negative
- Compensated liver disease
- Chronic ALT or AST elevation for 6 months prior to treatment
- Ishak score ≤ 2 (patients should only have mild fibrosis)
- Non cirrhotic
- No known active CMV infection
- Willingness to utilize adequate contraception while being treated with REP 9AC and for 6 months following the end of treatment
- Adequate venous access allowing weekly intravenous therapies and blood tests

Exclusion criterion (HBV + HCV patients)

- Evidence of cardiovascular disease
- Autoimmune hepatitis
- Presence of Wilson's disease
- Presence of severe NAFLD
- Evidence of any other co-existent liver disease
- ANA (anti-nuclear antibody): positive
- Anti-HIV 1: positive
- USG of hepato-biliary system: positive for cirrhosis of liver
- A history of ascites, hepatic encephalopathy or variceal hemorrhage
- Body weight > 100 kg
- Platelet count < 75,000, PMN count < 1,500 or HCT < 33%
- AFP > 100 ng/ml or the presence of a hepatic mass suggestive of HCC.
- Bilirubin > 2.5 mg/dl
- Creatinine > 1.5 mg/dl
- Platelets count < 75,000 / cmm
- Serum albumin < 35 mg/ml

- Poorly controlled diabetes mellitus
- Another serious medical disorder
- A serious psychiatric disorder
- Uncontrolled hypertension
- A history of alcohol abuse within the last year
- The use of illicit drugs within the past two years
- Inability to provide informed consent
- Positive pregnancy test
- Breastfeeding
- Inability or unwillingness to undergo a liver biopsy.
- Inability or unwillingness to provide weekly blood samples
- Poor venous access making weekly IV infusion too difficult

Patient enrollment

Once a patient has fulfilled all the criteria for enrollment, the data obtained and the enrollment submission form must be submitted to, and approved by REPLICor prior to entry into the trial.

Discontinuation of patient

The criterion for enrollment must be followed explicitly. In addition, patients will be discontinued from the drug study in the following circumstances:

> - If at any time during the study, a patient develops any conditions listed in the exclusion criterion, the principal investigator and REPLICor must be contacted to determine if patient discontinuation is warranted.
>
> - The principal investigator decides that the patient should be discontinued. If this decision is made because of a serious adverse event (SAE) or a clinically significant laboratory value, the study drug is to be stopped and appropriate measures are to be taken. A joint decision between the principal investigator and REPLICor will be made to decide if the patient can continue receiving the drug later on.
>
> - The patient is not capable or willing to continue participation in the study.

Dose escalation and dosing determination

It is unknown at what dosage, it will be possible to observe a reduction of viral titers in human patients receiving weekly intravenous administration of REP 9AC. In all animal studies using amphipathic polymers, therapeutic levels were reached before any sign of toxicity was noticeable. This study will use a dose escalation starting at 100 mg for the first week of active treatment and then moving up to 200 mg per week for the next 10 weeks, then to 400 mg per week for 10 weeks and then to 800 mg per week until a total of 40 weeks of active treatment has been completed . Because this is an adaptive trial, we may modify the dosing time periods as described below. Any modification to the

scheduled dosing will be done following discussion between the principal investigator and the sponsor always keeping the safety of the patient as the first priority.

The first week of treatment with an intravenous infusion, no active drug will be added to the 250 ml bag of normal saline. The patient **should be told** that he is receiving 100 mg of active compound on that week. The purpose is to make sure that

1- the patient can tolerate the amount of normal saline being administered
2- to understand what symptoms the patients may be experiencing in relation to the intravenous solution received and to understand that these symptoms **are not** drug related
3- to determine if a patient has a tendency to report symptoms purely based on his belief that he is receiving the drug.

The second week of treatment, only 100 mg of REP 9AC will be used in case a patient experiences an unforeseen reaction. The purpose is to minimize the potential reaction resulting from the administration. It is unlikely that we will see any bad reaction from such a low dose but the safety of the patient remains our first priority.

The patient will then start at 200 mg per week for the following 10 weeks. If a significant reduction of viral titers is noticed within this 10 week period, we may decide not to increase the dosage to the next dose level of 400 mg but instead to prolong the period of time that the patient will receive 200 mg per week to see if the titer reductions continue to improve. If the improvement is partial or if the decreases in titer stop improving, we will then go to the next dosing level at 400 mg per week.

The patient will then start at 400 mg per week for the following 10 weeks. If a significant reduction of viral titers is noticed within this 10 week period, we may decide not to increase the dosage to the next dose level of 800 mg but instead to prolong the period of time that the patient will receive 400 mg per week to see if the titer reductions continue to improve. If the improvement is partial or if the decreases in titer stop improving, we will then go to the next dosing level at 800 mg per week.

The patient will then start at 800 mg per week for the remaining duration of the study that will not exceed a total of 40 weeks of active treatment. In the event that the titer reductions continue to improve at the 40 week mark, we may submit to the IRB committee, a request to extend the duration of the treatment by submitting an investigator progress report.

If at any time during the duration of this trial, the viral titer of a patient becomes and remains undetectable, the dosage of the drug will be kept at the same level until a total of 40 weeks of active treatment has been given. No additional dose escalation will take place.

It is possible that we may choose to stop the dosing of selected patients (called sentinel patients) if their titers remain undetectable for a period of at least 12 weeks. This would be to assess what minimum amount of treatment may be necessary to reach a cure in responsive patients. These patients will be required to continue the monitoring process

on a weekly basis and would be started back on treatment using the last dose level as soon as the titers becomes measurable again. If they require re-treatment, they will continue treatment until they have received a total of 40 active treatments. If their titers remains undetectable, they will be followed for a minimum of 64 weeks from the start of the study like all other patients (40 weeks of treatments and 24 weeks of follow-up).

If a patient develops tolerable side effects, we may decide to stop the planned dose escalations and to maintain the patient on the same dose until a total of 40 weeks of treatment has been completed.

Missed doses

If a patient misses a treatment appointment, he should come in as soon as possible to receive the treatment that same week. Dosing appointments for subsequent weeks will not be altered.

If patient misses multiple treatments and it is decided to keep him on the study, these missed treatment can be added on past the scheduled end of treatment by extending the weekly treatment appointments by the number of treatments missed.

Adverse events

All adverse events must be promptly reported to REPLICor and joint decision will be made in collaboration with the principal investigator regarding any actions to be taken. The principal investigator will have the last word in any situation where there may be a disagreement with the sponsor. In every situation, the well-being of the patient shall remain the first priority.

The primary contact at REPLICor that should be contacted is:

Michel Bazinet, MD
mbazinet@replicor.com
(514) 951-6123 cellular
(450) 688-6068 ext-1 work

The second contact at REPLICor that should be contacted if Dr. Bazinet is not available:

Andrew Vaillant, Ph.D.
availlant@replicor.com
(514) 862-2271 cellular
(450) 688-6068 ext-3 work

Overview of study design

	Week on protocol	Visit number	Questionning	Abdominal exam	HBV / HCV serum titer PCR	HBV Antigens (E&S)	HBV seroconversion (E&S)	CBC	Serum tests	Liver Enzymes	PT & PTT	Urinalysis	Quality of Life Assessment	Drug Administration (mg)
Recruit			Safety		Efficacy			Safety						-
		1	X	X	X	X	X	X	X	X	X	X	X	-
		2	X	X										-
		3	X	X	X	X	X							-
Treatment	1	4	X	X				X		X	X	X	X	0
	2	5	X	X	X	X	X		X	X				100
	3	6	X	X	X			X		X	X			200
	4	7	X	X	X				X	X				200
	5	8	X	X	X			X		X	X	X	X	200
	6	9	X	X	X	X	X		X	X				200
	7	10	X	X	X			X		X	X			200
	8	11	X	X	X				X	X		X	X	200
	9	12	X	X	X			X		X	X			200
	10	13	X	X	X	X	X		X	X				200
	11	14	X	X	X			X		X	X			200
	12	15	X	X	X				X	X		X	X	200
	13	16	X	X	X			X		X	X			400
	14	17	X	X	X	X	X		X	X				400
	15	18	X	X	X			X		X	X			400
	16	19	X	X	X				X	X		X	X	400
	17	20	X	X	X			X		X	X			400
	18	21	X	X	X	X	X		X	X				400
	19	22	X	X	X			X		X	X			400
	20	23	X	X	X				X	X		X	X	400
	21	24	X	X	X			X		X	X			400
	22	25	X	X	X	X	X		X	X				400
	23	26	X	X	X			X		X	X			800
	24	27	X	X	X				X	X		X	X	800
	25	28	X	X	X			X		X	X			800
	26	29	X	X	X	X	X		X	X				800

		27	30		X	X		X				X		X	X				800
		28	31		X	X		X					X	X		X	X		800
		29	32		X	X		X				X		X	X				800
		30	33		X	X		X	X	X			X	X					800
		31	34		X	X		X				X		X	X				800
		32	35		X	X		X					X	X		X	X		800
		33	36		X	X		X				X		X	X				800
		34	37		X	X		X	X	X			X	X					800
		35	38		X	X		X				X		X	X				800
		36	39		X	X		X					X	X		X	X		800
		37	40		X	X		X				X		X	X				800
		38	41		X	X		X	X	X			X	X					800
		39	42		X	X		X				X		X	X				800
		40	43		X	X		X					X	X		X	X		800
		41	44		X	X		X				X		X	X				800
Follow-up		42	45		X	X		X	X	X			X	X					-
		44	46		X	X		X				X	X	X	X	X	X		-
		48	47		X	X		X	X	X		X	X	X	X	X	X		-
		52	48		X	X		X				X	X	X		X	X		-
		56	49		X	X		X	X	X		X	X	X		X	X		-
		60	50		X	X		X				X	X	X		X	X		-
		64	51		X	X		X	X	X		X	X	X		X	X		-

Material and supplies

The drug will be provided in borosilicate vials containing 1.1 ml of a solution with a concentration of 200 mg/ml. The vials will be identified with a label containing the following information:

Name: REP 2055 (REP 9AC)
Concentration: 200 mg/ml in 0.9 % NaCl
Volume: 1.1 ml
Date of fill finish

Each vial top has a red cover that must be intact until use to ensure sterility.

The drug should be stored in a refrigerator at between 4 ^{o} and 8 ^{o} degree Celsius until use. The drug can tolerate room temperature for a long period of time without degradation but it is recommended to keep it in optimal conditions to maximize its shelf life.

Prohibited concomitant therapies

Patients cannot received any drugs known to be active against HBV or HCV during the whole duration of the study. These drugs include interferon, ribavirin, protease inhibitors, polymerase inhibitors, nucleoside analogs, etc.

Concomitant therapies with restrictions on use

Patients should refrain from taking any drug that may have an impact on the liver, are known to carry a risk of hepatotoxicity or drugs that may by themselves generate unwanted side effects that could be interpreted as resulting from the administration of REP 9AC. Every week, the investigator will question the patient for any drug taken over the last 7 days.

Safety assessments to be performed

History review of any symptoms the patients may be reporting.

Abdominal examination including liver tenderness assessment.

To be assessed from blood drawn prior to REP 9AC IV infusion:

Hematology panel (CBC):

> WBC, RBC, Hemoglobin, Hematocryt, MCV, MCH, MCHC, RDW, Platelets count, MPV, Differential count and Sedimentation Rate

Clinical chemistry: serum (fasting for 14 hours) prior to treatment

> Sodium, Potassium, Chloride, Glucose, Calcium, Phosphorous, Uric Acid, BUN, Creatinine, Bilirubin total, direct and indirect, Globulin, Total Protein

Liver enzymes:

> Alkaline Phosphatase
> ALT
> AST
> GGT
> LDH

Cholesterol: total, triglycerides, HDL / LDL

PT / PTT

C-reactive protein

Urinalysis: Tested for heme & protein

Quality of life assessment questionnaire will be performed every 4 weeks using the standardized SF-36 questionnaire.

To be assessed at the beginning and at the end of the treatment cycle:

Liver pathology and TG content (if possible) from biopsy

Knodell necroinflammatory and fibrosis score from biopsy.

Biopsy assessment to include:

Fibrosis
Fat
Zone 3 pericellular fibrosis
Inflammation
Hepatocyte dysplasia
Bile duct lesions
Lymphoid follicles
Mallory bodies
Confluent necrosis
Hepatocellular iron grade.

To be assessed from frozen serum samples after study has been completed:

Serum apolipoprotein concentration: ApoB-48/100
ApoE (all isoforms)

Serum cytokine concentration: TNFα, INFα, IL-1β, IL-2, IL-4, IL-6, IL-8, IL-10, IP-10

Weekly urine to be held frozen for REP 9AC concentration determination at a later date.

Efficacy assessments to be performed

HBV infected patients:

- Serum HBV titer (PCR methodology to be determined).

- HBV antigenemia (S or E antigen) from stored blood samples

- HBeAg and HBsAg seroconversion

HCV infected patients:

Serum HCV titer (PCR methodology to be determined)

Changes in fibrotic state of livers will be assessed from liver biopsies at the beginning and the end of the study.

To be assessed from frozen serum samples after study is complete.

B-DNA titer assessment (HCV / HBV)

HCV core antigen ELISA

REP 9AC potentially induced mutations in HBV or HCV will be monitored by clonal sequencing if rebound is evident in any patient while receiving REP 9AC. Pheonotypic effects of mutations on susceptibility to REP 9AC activity will be assessed in vitro.

4. MONITORING AND DATA KEEPING

All the information collected during the trial about a specific patient will be collected in provided binders in the english language. Each patient will have a designated binder to collect the information obtained at each visit as well as the results of all the tests performed during the selection process as well as on a weekly basis.

All data obtained will be kept under the supervision of the principal investigator who will be responsible for the safekeeping of all the binders. In addition, as soon as practical, every new data generated will be scanned and e-mailed to the sponsor at the following address: results@ replicor.com

An external monitor may be assigned to the study by the sponsor. The monitor will receive the right to be present during the treatment of the patients on the study and to consult the binders of any patient he chooses. In addition, the monitor will be copied to all the information sent to results@replicor.com

Any results produced by outside laboratory should be sent concomitantly to the principal investigator, to REPLICor and to the monitor if applicable..

Principal investigator will be responsible for drug accountability on site.

5. INFORMED CONSENT

The principal investigator is responsible for ensuring that the patient understands the risks and benefits of participating in the study and should answer any questions the patient may have throughout the study in a timely manner. Also, the investigator must promptly inform the patient of important new developments that may impact the patient's willingness to continue participating in the study.

6. COSTS OF STUDY

All the costs incurred during the screening for inclusion and exclusion criteria during the selection process, the weekly laboratory evaluation and the biopsies will be covered by the sponsor. The drug will be provided for free for the duration of the trial.

7. REGULATORY CONSIDERATIONS

This study will be conducted in accordance with the ethical principles that are consistent with good clinical practice and the applicable laws and regulations of Bangladesh.

Clinical Trial Protocol
(Revised dosing regimen)

STUDY TITLE: Therapeutic safety and efficacy of REP 9AC in HBV or HCV infected patients.

PROTOCOL IDENTIFICATION NUMBER: REP101

STUDY SPONSOR: REPLICor Inc., Laval, Canada

INSTITUTION: Bangabandhu Sheikh Mujib Medical Center, Dhaka, Bangladesh

STUDY STATUS: IRB approved, study is active and in continued enrollment: two HBV patients in follow up, one patient (HCV) in active treatment.

STUDY DESIGN: Treatment, open label, safety / efficacy study, adaptive trial

ESTIMATED ENROLLMENT: 12 to 24 patients

ESTIMATED STUDY START DATE: January 2009 (patients on new dosing regimen anticipated to begin treatment in fall 2009)

STUDY AUTHORS: Mamun-Al-Mahtab, MD (Bangladesh), Principal Investigator
Andrew Vaillant, Ph.D. (REPLICor Inc.)
Michel Bazinet, MD (REPLICor Inc.)

1. INTRODUCTION

Chronic hepatitis B and C are long term conditions caused by infection of the body with the hepatitis B (HBV) and C (HCV) viruses. These infections often result in inflammation or scarring of the liver and can eventually lead to liver cirrhosis and liver failure. These infections are also one of the major causes of the development of hepatocellular carcinoma (liver cancer).

Hepatitis B. Although some drugs have been approved to treat chronic hepatitis B infections, they do not provide a complete cure except in rare cases (a cure generally means that a person loses the hepatitis B virus and develops protective surface antibodies). However, these drugs significantly decrease the risk of liver damage from the infection by slowing or stopping the virus from reproducing. Amongst the problems associated with currently available drugs are the tendency to develop resistance and the lack of clearance of the virus from the hepatocytes. There is clearly a need to identify

new drugs that can benefit patients with chronic hepatitis B infections. REP 9AC is a 40mer phosphorothioate oligonucleotide that has been shown to have low toxicity and to be highly effective to treat hepatitis B infection in animals. It has been shown to be effective in protecting animal from infection and to treat animals already infected.

The efficacy of REP 9AC in duck hepatitis B has been tested in collaboration with Dr. Allison Jilbert (University of Adelaide, Australia), who is a recognized expert on HBV infections. Our results in ducks suggest that our drug can completely eliminate the disease in a large proportion (50%) of animals following only 4 weeks of treatment. In other words, following cessation of the treatment, the viral titer does not return. If these results are replicated in humans, this will result in a paradigm shift in how patients with hepatitis B are treated. Currently available drugs can only control the disease. Once they are stopped, the viral titer returns.

This proposed study is designed to demonstrate that REP9AC can be well tolerated when given to human patients chronically infected with HBV and to evaluate if a reduction of viral titers can be observed when REP 9AC is administered as a monotherapy. Current interim data analysis from the REP 101 trial indicates the following:

1. REP 9AC is generally well tolerated at doses up to 600mg / week in all patients currently enrolled and in HBV patients at 400mg / day (for seven continuous days).

2. REP 9AC has resulted in both HBV patients achieving protective seroconversion (anti-HBs) and clearance of HBV DNA and HBsAg in their blood by 23 weeks after initiation of treatment.

3. Increasing frequency and dose of REP 9AC treatment at the start of therapy may provide more rapid seroconversion of HBV patients.

4. Increasing frequency and dose of REP 9AC treatment at the start of therapy may provide better efficacy for HCV patients.

Hepatitis C. Recent advances in the treatment of hepatitis C, using interferon and ribavirin combination therapy, have brought overall response rates to about 50% but these responses are associated with significant costs and toxicities. In view of the lack of response of many patients to standard therapy and in view of its associated toxicities, the development of new therapeutic strategies is critically important. Amphipathic polymers have been shown to have broad spectrum antiviral activities and to have very favorable pharmacokinetics in the liver making them ideal candidates for the therapy of viral hepatitis B and C.

In collaboration with Dr. Jake Liang, (Head, Liver Diseases Branch, National Institute of Health, USA), we have demonstrated that amphipathic DNA polymers are potent HCV entry inhibitors in vitro. In vivo, REP 9AC provides complete protection from HCV infection in naïve animals. REP 9AC is perfectly suited for the treatment of chronic HCV infection as exposure to this drug will allow the liver to naturally eliminate infected cells by halting the continual cycle of infection of healthy liver cells. REP 9AC may

eventually replace pegylated interferon and ribavirin as the standard of care owing to its activity against all genotypes of HCV and its novel mechanism of action, which does not promote the generation of drug resistance.

An entry inhibitor could be useful when used in combination therapy or could be effective as a monotherapy. This proposed study is designed to demonstrate that REP 9AC can be well tolerated when given to patients chronically infected with HCV and to evaluate if a reduction of viral titers can be observed when REP 9AC is administered as a monotherapy.

REP 9AC. REPLICor's technology utilizes the novel properties of modified oligonucleotides (phosphorothioate oligonucleotides) as amphipathic polymers to inhibit interactions critical for viral activity. This technology is active in vitro against all known families of enveloped viruses. REPLICor's proof of concept compounds, REP 9 and REP 9C and its lead compound, REP 9AC have also demonstrated potent antiviral activity in vivo against the following viral infections: HCV, HBV (DHBV), Cytomegalovirus, HSV-2, Ebola, influenza and respiratory syncytial virus.

REP 9, REP 9C and REP 9AC have been administered at therapeutically active doses in acute and chronic regimens by multiple routes of administration (parenteral, oral, topical and aerosol) in mice, rats, hamsters, guinea pigs, ducks and non human primate species with no detectable side effects. Moreover, this class of chemical compounds (phosphorothioate oligonucleotides) are known to have been well tolerated in human patients in several clinical trials.

REPLICor has verified the cGMP manufacture of REP 9AC which has been certified for human parenteral administration. The pharmacokinetic behavior of REP 9AC is similar to other compounds from the same chemical class (phosphorothioate oligonucleotides) which achieve long lasting, therapeutic liver concentrations typically using a single 200-400mg dose delivered once every week or once every other week in human patients.

2. OBJECTIVES

Primary objective

> To demonstrate that REP 9AC is well tolerated when given intravenously to patients infected with either chronic hepatitis B or C.

Secondary objective

> To evaluate the viral titer response following the intravenous administration of REP 9AC in patients infected with either chronic hepatitis B or C.

3. STUDY DESIGN AND SCHEDULE OF ASSESSMENTS

Treatment Period: Maximum of 40 weeks

Follow up: Minimum of 24 weeks (but scheduled for 57 weeks)

Dosing interval: First week: daily for seven continuous days.
Subsequent weeks: twice weekly

Dosing route: IV (20 hour slow infusion during daily administration, 180min infusion during twice weekly outpatient visits) with drug diluted in 250cc normal saline

Dose level: 400mg

Number of patients: 12 to 24

Patient inclusion criterion (HBV)

- Age between 18 and 55
- HBsAg+ for at least 6 months prior to initiation of treatment.
- HBeAg+
- HBcAg IgM > 1000 U/L
- HBV titer > 20000 copies/ml at start of treatment
- Treatment naïve
- HIV/HDV/HCV negative
- Compensated liver disease
- Ishak score ≤ 2
- Non cirrhotic
- No known active CMV infection
- Willingness to utilize adequate contraception while being treated with REP 9AC and for 6 months following the end of treatment
- Adequate venous access allowing weekly intravenous therapies and blood tests

Patient inclusion criterion (HCV)

- Age between 18 and 55
- HCV positive for at least 6 months prior to initiation of treatment
- Genotype 3
- HCV titer >3log IU/ml at start of treatment
- Treatment naïve
- HIV/HDV/HBV negative
- Compensated liver disease
- Chronic ALT or AST elevation for 6 months prior to treatment
- Ishak score ≤ 2 (patients should only have mild fibrosis)
- Non cirrhotic

- No known active CMV infection
- Willingness to utilize adequate contraception while being treated with REP 9AC and for 6 months following the end of treatment
- Adequate venous access allowing weekly intravenous therapies and blood tests

Exclusion criterion (HBV + HCV patients)

- Evidence of cardiovascular disease
- Autoimmune hepatitis
- Presence of Wilson's disease
- Presence of severe NAFLD
- Evidence of any other co-existent liver disease
- ANA (anti-nuclear antibody): positive
- Anti-HIV 1: positive
- USG of hepato-biliary system: positive for cirrhosis of liver
- A history of ascites, hepatic encephalopathy or variceal hemorrhage
- Body weight > 100 kg
- Platelet count < 75,000, PMN count < 1,500 or HCT < 33%
- AFP > 100 ng/ml or the presence of a hepatic mass suggestive of HCC.
- Bilirubin > 2.5 mg/dl
- Creatinine > 1.5 mg/dl
- Platelets count < 75,000 / cmm
- Serum albumin < 35 mg/ml
- Poorly controlled diabetes mellitus
- Another serious medical disorder
- A serious psychiatric disorder
- Uncontrolled hypertension
- A history of alcohol abuse within the last year
- The use of illicit drugs within the past two years
- Inability to provide informed consent
- Positive pregnancy test
- Breastfeeding
- Inability or unwillingness to undergo a liver biopsy.
- Inability or unwillingness to provide weekly blood samples
- Poor venous access making weekly IV infusion too difficult

Patient enrollment

Once a patient has fulfilled all the criteria for enrollment, the data obtained and the enrollment submission form must be submitted to, and approved by REPLICor prior to entry into the trial.

Discontinuation of patient

The criterion for enrollment must be followed explicitly. In addition, patients will be discontinued from the drug study in the following circumstances:

- If at any time during the study, a patient develops any conditions listed in the exclusion criterion, the principal investigator and REPLICor must be contacted to determine if patient discontinuation is warranted.

- The principal investigator decides that the patient should be discontinued. If this decision is made because of a serious adverse event (SAE) or a clinically significant laboratory value, the study drug is to be stopped and appropriate measures are to be taken. A joint decision between the principal investigator and REPLICor will be made to decide if the patient can continue receiving the drug later on.

- The patient is not capable or willing to continue participation in the study.

Early entry into follow up

It is possible that we may choose to stop the dosing of selected patients (called sentinel patients) if they show evidence of having achieved protective levels of anti-HBs in their blood. These patients will be required to continue the follow up monitoring process as scheduled and would be started back on treatment only if they show sign of a relapse which they cannot self resolve. If they require re-treatment, they will repeat the week of daily drug administration, followed by twice weekly drug administration until they have cleared HBV DNA and HBsAg.

Missed doses

If a patient misses a treatment appointment, he should come in as soon as possible to receive the treatment that same week. Dosing appointments for subsequent weeks will not be altered.

If patient misses multiple treatments and it is decided to keep him on the study, these missed treatment can be added on past the scheduled end of treatment by extending the weekly treatment appointments by the number of treatments missed.

Adverse events

All adverse events must be promptly reported to REPLICor and joint decision will be made in collaboration with the principal investigator regarding any actions to be taken. The principal investigator will have the last word in any situation where there may be a disagreement with the sponsor. In every situation, the well-being of the patient shall remain the first priority.

The primary contact at REPLICor that should be contacted is:

Michel Bazinet, MD
mbazinet@replicor.com
(514) 951-6123 cellular
(450) 688-6068 ext-1 work

The second contact at REPLICor that should be contacted if Dr. Bazinet is not available:

Andrew Vaillant, Ph.D.
availlant@replicor.com
(514) 862-2271 cellular
(450) 688-6068 ext-3 work

Overview of study design

	Week on protocol	Questionning	Abdominal exam	HBV / HCV serum titer PCR	HBsAg	seroconversion (anti-HBs)	ECG	CBC	Serum tests	Liver Enzymes	PT & PTT	Urinalysis	Quality of Life Assessment	Drug Administration (mg)
		Safety		Efficacy			Safety							
Recruit		X	X	X	X	X	X	X	X	X	X	X	X	0
		X	X											0
		X	X	X	X	X								0
Treatment	1 d1	X	X	X	X	X	X	X	X	X	X	X	X	400
	1 d2	X	X	X	X	X	X	X	X	X	X	X		400
	1 d3	X	X								X			400
	1 d4	X	X								X			400
	1 d5	X	X	X	X	X	X	X	X	X	X	X		400
	1 d6	X	X								X			400
	1 d7	X	X								X			400
	2	X	X	X	X	X	X	X	X	X	X	X	X	0
	2 d4													400
	3	X	X	X	X	X	X	X		X	X	X		400
	3 d4													400
	4	X	X	X	X	X	X		X	X	X	X	X	400
	4 d4													400
	5	X	X	X	X	X	X	X		X	X	X		400
	5 d4													400
	6	X	X	X	X	X	X		X	X	X	X		400
	6 d4													400
	7	X	X	X	X	X	X	X		X	X	X		400
	7 d4													400
	8	X	X	X	X	X	X		X	X	X	X	X	400
	8 d4													400
	9	X	X	X	X	X	X	X		X	X	X		400

		9 d4																	400
		10		X	X		X	X	X		X		X	X	X	X			400
		10 d4																	400
		11		X	X		X	X	X		X	X		X	X	X			400
		11 d4																	400
		12		X	X		X	X	X		X		X	X	X	X	X		400
		12 d4																	400
		13		X	X		X	X	X		X	X		X	X	X			400
		13 d4																	400
		14		X	X		X	X	X		X		X	X	X	X			400
		14 d4																	400
		15		X	X		X	X	X		X	X		X	X	X			400
		15 d4																	400
		16		X	X		X	X	X		X		X	X	X	X	X		400
		16d4																	400
		17		X	X		X	X	X		X	X		X	X	X			400
		17 d4																	400
		18		X	X		X	X	X		X		X	X	X	X			400
		18 d4																	400
		19		X	X		X	X	X		X	X		X	X	X			400
		19 d4																	400
		20		X	X		X	X	X		X		X	X	X	X	X		400
		20 d4																	400
		21		X	X		X	X	X		X	X		X	X	X			400
		21 d4																	400
		22		X	X		X	X	X		X		X	X	X	X			400
		22 d4																	400
		23		X	X		X	X	X		X	X		X	X	X			400
		23 d4																	400
		24		X	X		X	X	X		X		X	X	X	X	X		400
		24 d4																	400
		25		X	X		X	X	X		X	X		X	X	X			400
		25 d4																	400
		26		X	X		X	X	X		X		X	X	X	X			400
		26 d4																	400
		27		X	X		X	X	X		X	X		X	X	X			400
		27 d4																	400
		28		X	X		X	X	X		X		X	X	X	X	X		400
		28 d4																	400
		29		X	X		X	X	X		X	X		X	X	X			400
		29 d4																	400
		30		X	X		X	X	X		X		X	X	X	X			400
		30 d4																	400
		31		X	X		X	X	X		X	X		X	X	X			400
		31 d4																	400
		32		X	X		X	X	X		X		X	X	X	X	X		400

		32 d4																400
		33		X	X		X	X	X		X	X		X	X	X			400
		33 d4																400
		34		X	X		X	X	X		X		X	X	X	X			400
		34 d4																400
		35		X	X		X	X	X		X	X		X	X	X			400
		35 d4																400
		36		X	X		X	X	X		X		X	X	X	X	X		400
		36 d4																400
		37		X	X		X	X	X		X	X		X	X	X			400
		37 d4																400
		38		X	X		X	X	X		X		X	X	X	X			400
		38 d4																400
		39		X	X		X	X	X		X	X		X	X	X			400
		39 d4																400
		40		X	X		X	X	X		X		X	X	X	X	X		400
Follow-up		41		X	X		X	X	X		X	X	X	X	X	X			0
		43		X	X		X	X	X		X	X	X	X	X	X			0
		45		X	X		X	X	X		X	X	X	X	X	X	X		0
		47		X	X		X	X	X		X	X	X	X	X	X			0
		49		X	X		X	X	X		X	X	X	X	X	X	X		0
		53		X	X		X	X	X		X	X	X	X	X	X	X		0
		57		X	X		X	X	X		X	X	X	X	X	X	X		0
		65		X	X		X	X	X		X	X	X	X	X	X	X		0
		81		X	X		X	X	X		X	X	X	X	X	X	X		0
		97		X	X		X	X	X		X	X	X	X	X	X	X		0

Material and supplies

The drug will be provided in borosilicate vials containing 1.1 ml of a solution with a concentration of 200 mg/ml. The vials will be identified with a label containing the following information:

Name: REP 2055 (REP 9AC)
Concentration: 200 mg/ml in 0.9 % NaCl
Volume: 1.1 ml
Date of fill finish

Each vial top has a red cover that must be intact until use to ensure sterility.

The drug should be stored in a refrigerator at between 4 ^{o} and 8 ^{o} degree Celsius until use. The drug can tolerate room temperature for a long period of time without degradation but it is recommended to keep it in optimal conditions to maximize its shelf life.

Supplementation

Recent reports now provide evidence of substantial vitamin D deficiency (Fisher and Fisher 2007 Clin Gastro. Hepatol. 2007 5: 513-520) and dysfunction in mineral homeostasis (George J. et al. World J. Gastroenterol. 2009 15: 3516-3522)- in patients with chronic liver disease. In order to ensure the well being of all future patients being enrolled on to the REP 101 study, patients will receive a daily, over the counter mineral / vitamin D3 supplement taken orally each day while they are receiving REP 9AC.

Prohibited concomitant therapies

Patients cannot receive any drugs known to be active against HBV or HCV during the whole duration of the study. These drugs include interferon, ribavirin, protease inhibitors, polymerase inhibitors, nucleoside analogs, etc.

Concomitant therapies with restrictions on use

Patients should refrain from taking any drug that may have an impact on the liver, are known to carry a risk of hepatotoxicity or drugs that may by themselves generate unwanted side effects that could be interpreted as resulting from the administration of REP 9AC. Every week, the investigator will question the patient for any drug taken over the last 7 days.

Safety assessments to be performed

History review of any symptoms the patients may be reporting.

Abdominal examination including liver tenderness assessment.

To be assessed from blood drawn prior to REP 9AC IV infusion:

Hematology panel (CBC):

Hemoglobin, Platelets count, Sedimentation Rate, WBC and differential count.

Clinical chemistry: serum, prior to treatment

Sodium, Potassium, Chloride, Glucose, Calcium, FBS, Uric Acid, Creatinine, Bilirubin, Albumin, Globulin and Total Protein.

Liver enzymes:

Alkaline Phosphatase
SGPT (ALT)
SGOT (AST)

Cholesterol: total, triglycerides, HDL, LDL

PT / PTT

Urinalysis: Tested for heme & protein

Quality of life assessment questionnaire will be performed every 4 weeks using the standardized SF-36 questionnaire.

To be assessed at the beginning and at the end of the treatment cycle:

Liver pathology and TG content (if possible) from biopsy

Knodell necroinflammatory and fibrosis score from biopsy.

Biopsy assessment to include:

Fibrosis
Fat
Zone 3 pericellular fibrosis
Inflammation
Hepatocyte dysplasia
Bile duct lesions
Lymphoid follicles
Mallory bodies
Confluent necrosis
Hepatocellular iron grade.

To be assessed from frozen serum samples after study has been completed:

Serum apolipoprotein concentration:	ApoB-48/100<br>ApoE (all isoforms)
Serum cytokine concentration:	TNFα, INFα, IL-1β, IL-2, IL-4, IL-6, IL-8, IL-10, IP-10

Quantitative HBsAg determination using Architect ™ ELISA

Weekly urine to be held frozen for REP 9AC concentration determination at a later date.

Efficacy assessments to be performed

HBV infected patients:

- Serum HBV titer (PCR methodology to be determined) from fresh samples.

- HBV antigenemia (S antigen) from fresh and frozen serum samples

- HBsAg seroconversion from fresh samples

HCV infected patients:

Serum HCV titer (PCR methodology to be determined) from fresh samples

Changes in fibrotic state of livers will be assessed from liver biopsies at the beginning and the end of the study.

To be assessed from frozen serum samples after study is complete.

B-DNA titer assessment (HCV / HBV)

HCV core antigen ELISA

Any mutations induced by REP 9AC in HBV or HCV may be monitored by clonal sequencing if rebound is evident in any patient while receiving REP 9AC. Pheonotypic effects of mutations on susceptibility to REP 9AC activity will be assessed in vitro.

4. MONITORING AND DATA KEEPING

All the information collected during the trial about a specific patient will be collected in provided binders in the english language. Each patient will have a designated binder to collect the information obtained at each visit as well as the results of all the tests performed during the selection process as well as on a weekly basis.

All data obtained will be kept under the supervision of the principal investigator who will be responsible for the safekeeping of all the binders. In addition, as soon as practical, every new data generated will be scanned and e-mailed to the sponsor at the following addresses: mbazinet@replicor.com and availlant@replicor.com

An external monitor may be assigned to the study by the sponsor. The monitor will receive the right to be present during the treatment of the patients on the study and to consult the binders of any patient he chooses. In addition, the monitor will be copied to all the information sent to results@replicor.com

Any results produced by outside laboratory should be sent concomitantly to the principal investigator, to REPLICor and to the monitor if applicable..

Principal investigator will be responsible for drug accountability on site.

5. INFORMED CONSENT

The principal investigator is responsible for ensuring that the patient understands the risks and benefits of participating in the study and should answer any questions the patient may have throughout the study in a timely manner. Also, the investigator must promptly

inform the patient of important new developments that may impact the patient's willingness to continue participating in the study.

6. COSTS OF STUDY

All the costs incurred during the screening for inclusion and exclusion criteria during the selection process, the weekly laboratory evaluation and the biopsies will be covered by the sponsor. The drug will be provided for free for the duration of the trial.

7. REGULATORY CONSIDERATIONS

This study will be conducted in accordance with the ethical principles that are consistent with good clinical practice and the applicable laws and regulations of Bangladesh.
