# Supplementary material for: Safety and Efficacy of Nucleic Acid Polymers in Monotherapy and Combined with Immunotherapy in Treatment-Naive Bangladeshi Patients with HBeAg+ Chronic Hepatitis B Infection
Source: PLoS One. 2016 Jun 3;11(6):e0156667. doi: 10.1371/journal.pone.0156667 (PMC4892580; doi:10.1371/journal.pone.0156667)
Supplement: S3 File — (PDF) [file pone.0156667.s003.pdf]

Clinical Trial Protocol

STUDY TITLE: Therapeutic safety and efficacy of REP 2139 (REP 9AC’) in HBV infected patients.

PROTOCOL IDENTIFICATION NUMBER: REP 102

STUDY SPONSOR: REPLICor Inc., Laval, Canada

INSTITUTION: Bangabandhu Sheikh Mujib Medical Center, Dhaka, Bangladesh

STUDY STATUS: awaiting IRB approval

STUDY DESIGN: Treatment, open label, safety / efficacy study, adaptive trial

ESTIMATED ENROLLMENT: 8-12 patients

ESTIMATED STUDY START DATE: August 2011

STUDY AUTHORS: Mamun-Al-Mahtab, MD (Bangladesh), Principal Investigator
Andrew Vaillant, Ph.D. (REPLICor Inc.)
Michel Bazinet, MD (REPLICor Inc.)

1. INTRODUCTION

Chronic hepatitis B is a long term condition caused by infection of the body with the hepatitis B virus (HBV). This infection often results in inflammation or scarring of the liver and can eventually lead to liver cirrhosis and liver failure. These infections are also one of the major causes of the development of hepatocellular carcinoma (liver cancer).

Although some drugs have been approved to treat chronic hepatitis B infections, they do not provide a complete cure except in rare cases (a cure generally means that a person loses the hepatitis B virus from the blood and the liver and develops a durable immunological control of subsequent HBV infection). However, these drugs do significantly decrease the risk of liver damage and liver cancer arising from the presence of a chronic liver infection by slowing or stopping the production of infectious virus. Thus the primary problem associated with currently available drugs is the lack of clearance of the virus from the hepatocytes which necessitates long term treatment with these drugs. There is clearly a need to identify new drugs that can benefit patients with chronic hepatitis B infections. Nucleic acid-bases polymers (NAPs) are a new class of broad-spectrum antiviral compounds which act against HBV infection by blocking the

release of the surface antigen protein (HBsAg) from infected hepatocytes. In the human patients in the REP 101 protocol, the previous NAP clinical candidate, REP 9AC (REP 2055), rapidly induced pronounced reductions in or clearance of serum HBsAg in 7 out of 8 patients. HBsAg is the major immunoinhibitory mechanism by which HBV maintains its chronicity and by reducing or eliminating HBsAg from the blood, REP 9AC appears able to elicit restoration of durable immunological responses in patients capable of clearing the HBV infection. The performance of REP 9AC in providing sustained virologic responses (SVRs) in patients with chronic HBV infection appeared to be far superior than any compound currently approved for the treatment of HBV infection

REP 9AC' (REP 2139) is a modified version of its predecessor, REP 9AC. Both are 40mer phosphorothioate oligonucleotides comprised of alternating adenosine and cytidine nucleotides and in the case of REP 9AC, has been shown to have low toxicity and to be highly effective in treating hepatitis B infection in human patients. The modifications in REP 9AC' significantly improve the stability and reduce the pro-inflammatory activity of REP 9AC' compared to that of REP 9AC while retaining all the antiviral activity found in REP 9AC. Both these modifications (5-methylation of cytosines and 2' O methylation of the ribose sugar in each nucleotide) are naturally occurring modifications in human nucleic acid and are known to be well tolerated in clinical trials. It is expected that REP 9AC' will be able to achieve a more robust antiviral activity in patients with chronic HBV with significantly lower dosing requirements and fewer side effects than REP 9AC.

Current interim data analysis from the still ongoing REP 101 assessing the activity of REP 9AC in patients with chronic HBV infection indicates the following:

1. REP 9AC exposure in eight patients has been generally well tolerated at doses up to 600mg / week and 400mg / day (for seven continuous days). Administration related side effects include mild to moderate pro-inflammatory reactions during the drug administration (itching and fever) which disappear after drug administration is complete. Chronic side effects include mild elevations in INR (~1.5 X) and serum hypocalcemia (which is easily mitigated with a mineral supplement).

2. REP 9AC has achieved serum HBsAg reduction or clearance in all patients compliant with the proscribed dosing regimen (7 out of 7).

3. In these seven patients, 5 have demonstrated restoration of at least a partial immunological control of their infection (appearance of serum anti-HBs and the establishment of a lower serum HBV DNA setpoint).

4. Of the five patients achieving having an immunological response, three have achieved a SVR off treatment with as few as 20 weeks of REP 9AC treatment with complete and durable immunological control over their infection for 18, 12 and 10.5 months.

5. Suboptimal stability of REP 9AC is a likely factor impairing the performance of the drug.

This proposed study is designed to demonstrate that REP 9AC’ can be well tolerated when given to human patients chronically infected with HBV and to evaluate if a reduction of viral titers can be observed when REP 9AC’ is administered as a monotherapy.

NAPs: REPLICor’s technology utilizes the novel properties of NAPs to inhibit interactions critical for viral activity. This technology is active in vitro against all known families of enveloped viruses. REPLICor’s proof of concept compounds, REP 9 and REP 9C and its first generation clinical candidate, REP 9AC have also demonstrated potent antiviral activity in vivo against the following viral infections: HCV, HBV (DHBV), Cytomegalovirus, HSV-2, Ebola, influenza and respiratory syncytial virus.

NAPs (REP 9, REP 9C and REP 9AC) have been administered at therapeutically active doses in acute and chronic regimens by multiple routes of administration (parenteral, oral, topical and aerosol) in mice, rats, hamsters, guinea pigs, ducks and non human primate species with no detectable side effects. Moreover, this class of chemical compounds (phosphorothioate oligonucleotides) are known to be well tolerated in human patients in several clinical trials.

REPLICor has validated the compatibility of the modifications in REP 9AC’ with the antiviral activity present in NAPs indirectly: the 2’O methyl sugar modification was found to not affect the antiviral activity of NAPs against duck hepatitis B virus in vivo and further shown to substantially improve the stability of NAPs to nuclease degradation and substantially reduce their immunoreactivity in human PBMCs. The 5’methylation of cytosine was shown not to affect target interaction in cell free interaction assays and is well known to mitigate the immunoreactivity of nucleic acids.

REPLICor has verified the cGMP manufacture of REP 9AC’ which will be certified for human parenteral administration. The biodistrubution of REP 9AC’ is similar to other compounds from the same chemical class (phosphorothioate oligonucleotides) which achieve long lasting, therapeutic liver concentrations typically using a single 200-400mg dose delivered once every week or once every other week in human patients. However, it is expected that REP 9AC’ will not suffer from the degradation occurring in REP 9AC (shortening of the polymer from the ends) which will result in fully active compound being present for a much longer time.

2. OBJECTIVES

Primary objective

> To demonstrate that REP 9AC’ is well tolerated when given intravenously or subcutaneously to patients infected with chronic hepatitis B.

Secondary objective

To evaluate the effect of REP 9AC' administration on the reduction of serum HBsAg and subsequent restoration of immunological control over chronic HBV infection (the detection of anti-HBsAg antibodies and reduction or clearance of serum HBV DNA).

3. STUDY DESIGN AND SCHEDULE OF ASSESSMENTS

Treatment Period: Scheduled for 40 weeks

Follow up: Minimum of 52 weeks.

Dosing interval: First week: daily for seven continuous days (IV).
Subsequent weeks **(optional – if serum HBsAg is not cleared)**: once or twice weekly (IV or SC)

Dosing route: IV: 5 - 20 hour slow infusion during daily administration, 5 – 20 hour infusion during once or twice weekly outpatient visits with drug diluted in 250cc normal saline

SC: one or two (contralateral) bolus injections (of one or two cc's)

Dose level: Drug product is prepared as a 25mg/ml calcium chelate of REP 9AC' in normal saline (2cc in a 3cc syringe). IV infusions will range from 250-1000mg daily, given in rounds of 250mg REP 9AC' in 250cc normal saline.

SC injections will range from 25mg (1cc) to 50mg (2cc) once weekly (typically given after the IV infusion loading phase if necessary).

Number of patients: 8-12

Patient inclusion criterion

- Age between 18 and 55
- HBsAg+
- Anti-HBs negative
- HBV titer > $1x10^7$ copies / ml
- Treatment naïve
- HIV/HCV/HDV negative
- Fibrosis with compensation (as determined by Fibroscan and liver enzymes)
- Non cirrhotic
- No known active CMV infection
- Willingness to utilize adequate contraception while being treated with REP 9AC and for 6 months following the end of treatment
- Adequate venous access allowing weekly intravenous therapies and blood tests

Exclusion criterion

- Evidence of cardiovascular disease
- Autoimmune hepatitis
- Presence of Wilson's disease
- Presence of severe NAFLD
- Evidence of any other co-existent liver disease
- ANA (anti-nuclear antibody): positive
- USG of hepato-biliary system: positive for cirrhosis of liver
- A history of ascites, hepatic encephalopathy or variceal hemorrhage
- Body weight > 100 kg
- Platelet count < 75,000, PMN count < 1,500 or HCT < 33%
- AFP > 100 ng/ml or the presence of a hepatic mass suggestive of HCC.
- Bilirubin > 2.5 mg/dl
- Creatinine > 1.5 mg/dl
- Platelet count < 75,000 / cmm
- Serum albumin < 35 mg/ml
- Poorly controlled diabetes mellitus
- Another serious medical disorder
- A serious psychiatric disorder
- Uncontrolled hypertension
- A history of alcohol abuse within the last year
- The use of illicit drugs within the past two years
- Inability to provide informed consent
- Positive pregnancy test
- Breastfeeding
- Inability or unwillingness to provide weekly blood samples
- Poor venous access making IV infusion too difficult

Patient Screening and Surveillance

Patients will be identified as prospective candidates and entered into a surveillance of their viral infection on the basis of a preliminary HBV DNA test (which must show a serum HBV DNA ≥ 10^6 cpm. Patients will be subjected to a minimum of three surveillance visits where their viremia and fibrosis state will be assessed. Patients will also be asked to take a standard multivitamin and mineral supplement during the surveillance phase.

Viremia Screening and Surveillance Procedure for REP 102 Patient Enrollments

Monitoring	Virologic tests	NOTES
Screening	HBV DNA	HBV DNA ≥ 10^7 to enter into surveillance
Surveillance WK1	HBV DNA, HBsAg, HBeAg, anti-HBs,	Multivitamin and mineral supplements started
Surveillance WK5	HBV DNA, HBsAg, anti-HBs, Fibroscan	Informed consent signed, serum samples retained
Surveillance WK9	HBV DNA, HBsAg, anti-HBs	Serum samples retained
Final assessment* (week prior to first compound administration)	HBV DNA (+ genotyping), HBsAg, anti-HBs	Serum samples retained

*includes all testing to verify patient meets inclusion and passes exclusion criteria.

Patient enrollment

Once a patient has fulfilled all the criteria for enrollment, the data obtained and the enrollment submission form must be submitted to, and approved by REPLICor prior to a patient being entered into the trial. Each patient entered into the trial will receive a unique patient identifier (REP 102-X)

Management of REP 9AC' dosing

Several patients in the REP 101 protocol (REP 101-02B, 04B and 08B) rapidly cleared their serum HBsAg with 400-800mg of REP 9AC given in seven daily or every other day doses. Because of the substantially improved stability of REP 9AC' over REP 9AC, it is expected that patients receiving REP 9AC' IV infusions will benefit from a rapid and very long lasting suppression of their serum HBsAg. Therefore, patients clearing their serum HBsAg during the first week of treatment will come to the clinic during the subsequent weeks only for physical examination and blood testing but no drug will be administered until the HBsAg becomes detectable. If and when the HBsAg becomes detectable, patient will be given one additional gram of REP 9AC' by intravenous infusion which will be followed by weekly administration of 50 mg of REP 9AC' given subcutaneously. If patients have not completely cleared their serum HBsAg during the first week of treatment (but tolerated the infusions well), they will receive a second and potentially a third intravenous administration on an outpatient basis during the following two weeks. If additional REP 9AC' dosing is required to clear HBsAg or maintain HBsAg seroclearance, it will be administered by subcutaneous injection.

If a patient clears their serum HBsAg during the first week and this clearance is durable for several weeks after with no additional dosing, future serum HBsAg rebounds in this patient may be treated with outpatient IV infusions of REP 9AC' with longer intervals between doses.

Discontinuation of REP 9AC' treatment

The criterion for enrollment must be followed explicitly. In addition, patients will be discontinued from the drug study in the following circumstances:

- If at any time during the study, a patient develops any conditions listed in the exclusion criterion, the principal investigator and REPLICor must be contacted to determine if patient discontinuation is warranted.

- The principal investigator decides that the patient should be discontinued. If this decision is made because of a serious adverse event (SAE) or a clinically significant laboratory value, the study drug is to be stopped and appropriate measures are to be taken. A joint decision between the principal investigator and REPLICor will be made to decide if the patient can continue receiving the drug later on.

- The patient is not capable or willing to continue participation in the study.

- The patient is non-compliant with the proscribed dosing and supplementation protocol.

- The patient meets the performance criteria (see below) for early entry into followup.

Rules for early entry into followup

Based on performance data from the REP 101 protocol, the following performance criterion for patients on REP 9AC' monotherapy will be used to identify patients which have demonstrated complete control over their infection and in which durable immunological control is likely to persist after treatment is withdrawn.

At least four continuous weeks of HBV DNA < 500 cpm

Patients meeting this criteria at any time after starting REP 9AC' monotherapy may be permitted to stop REP 9AC' treatment and patients admitted early into the follow up phase must follow the monitoring process as scheduled and would be started back on treatment only if they show signs of a relapse which they cannot self-resolve.

Missed doses

If a patient misses a treatment appointment, he should come in as soon as possible to receive the treatment that same week. Dosing appointments for subsequent weeks will not be altered.

If patient misses multiple treatments and it is decided to keep him on the study, these missed treatments can be added on past the scheduled end of treatment by extending the weekly treatment appointments by the number of treatments missed.

Adverse events

All adverse events must be promptly reported to REPLICor and a joint decision will be made in collaboration with the principal investigator regarding any actions to be taken. The principal investigator will have the last word in any situation where there may be a disagreement with the sponsor. In every situation, the well-being of the patient shall remain the first priority.

The primary contact at REPLICor that should be contacted is:

Michel Bazinet, MD
mbazinet@replicor.com
(514) 951-6123 cellular

(514) 496-9016 work

The second contact at REPLICor that should be contacted if Dr. Bazinet is not available:

Andrew Vaillant, Ph.D.
availlant@replicor.com
(514) 862-2271 cellular
(514) 496-9011 work

Overview of study design

Initial Dosing:

Patients	Day 1	Day 2	Day 3	Day 4	Day 5
01 – 02	500 mg<br>8 hrs	500 mg<br>8 hrs	1 g<br>8 hrs	1g<br>8 hrs	1g<br>8 hrs
03 – 04	1g<br>8 hrs	1 g<br>8 hrs	2 g<br>8 hrs		
05 – 06	2 g<br>8 hrs	2 g<br>4 hrs			
07 - 08	4 g<br>8 hrs				

Note: One human has already received over 4 grams of REP 9AC' by intravenous infusion with no detectable side effects.

Efficacy Measurements

Test	Interval
Fibroscan	Surveillance WK1, 12 months follow up
Serum HBV DNA (ROCHE COBAS)	Screening, surveillance and weekly during treatment
HBsAg (Immunilite)	Screening, surveillance, daily during first week and weekly thereafter
Anti-HBs (Immunilite)	Screening, surveillance, daily during first week and weekly thereafter
HBeAg (Immunilite)	1st surveillance visit and weekly after HBV DNA < 500cpm until withdrawl from REP 9AC' treatment
Anti-HBe (Immunilite)	weekly after HBV DNA < 500cpm until withdrawl from REP 9AC' treatment

Safety Measurements (see also section on safety assessments below)

Test	Interval
CBC	Weekly for 4 weeks then transitioning to once a month if no adverse events are observed
PT/PTT	Daily for the first week then following schedule for hematology
Liver enzymes	Daily for the first week then following schedule for hematology
Clinical chemistry	Same as hematology
Fasting cholesterol	Same as PT/PTT
ECG	Once monthly
Urinalysis	Weekly

Material and supplies

Subcutaneous preparations

The drug will be provided in 3cc polycarbonate syringes prefilled with 2.1cc of 25mg/ml REP 9AC calcium chelate. The syringes will be identified with a label containing the following information:

Name: REP 2139•Ca 25mg/ml
Date of fill finish

Each syringe has a luer lock cap that must be intact until use to ensure sterility. Each syringe is enclosed in a sterile plastic envelop that should only be opened at the time of administration.

Intravenous solutions

The drug will be provided in pre-filled sterile normal saline bags indicating the name of the drug and the total dose contained in the bag. These intravenous solutions will be ready to administer. The recommended speed of administration will be provide to the principal investigator for each patient. Each bag will have a label with the following information:

Name: REP 2139•Ca
Dose in mg
Date of fill finish

Each bag will be enclosed in a sterile plastic envelope that should only be opened at the time of administration. DEHP tubing for intravenous administration will be provided.

Drug storage

The drug should be stored in a refrigerator at between 4° and 8° degrees Celsius until use. The drug can tolerate room temperature for a long period of time without degradation but it is recommended to keep it in optimal conditions to maximize its shelf life.

Mineral Supplementation

Recent reports now provide evidence of substantial vitamin D deficiency (Fisher and Fisher 2007 Clin Gastro. Hepatol. 2007 5: 513-520) and dysfunction in mineral homeostasis (George J. et al. World J. Gastroenterol. 2009 15: 3516-3522)- in patients with chronic liver disease. In order to ensure the well-being of all future patients being enrolled on to the REP 102 study, patients will receive a daily, over the counter multivitamin and mineral / vitamin D3 supplement taken orally each day while they are participating in the REP 102 study.

Prohibited concomitant therapies

Patients cannot receive any drugs known to be active against HBV or HCV during the whole duration of the study. These drugs include interferon, ribavirin, protease inhibitors, polymerase inhibitors, nucleoside analogs, etc.

Concomitant therapies with restrictions on use

Patients should refrain from taking any drug other than REP 9AC' unless specifically authorized by the principal investigator. Every week, the investigator will question the patient for any drug taken over the last 7 days.

Safety assessments to be performed

History review of any symptoms the patients may be reporting.

Abdominal examination including liver tenderness assessment.

To be assessed from blood drawn prior to REP 9AC IV infusion:

Hematology panel (CBC):

> Hemoglobin, Platelets count, Sedimentation Rate, WBC and differential WBC count, RBC count and RBC indices.

Clinical chemistry: serum, prior to treatment

> Sodium, Potassium, Chloride, Glucose, Calcium, Magnesium, Zinc, Copper, Uric Acid, Creatinine, Bilirubin, Albumin, Globulin and Total Protein.

Liver enzymes:

Alkaline Phosphatase
SGPT (ALT)
SGOT (AST)

Cholesterol: total, triglycerides, HDL, LDL

PT / PTT

Urinalysis: Tested for heme & protein

4. MONITORING AND DATA KEEPING

All the information collected during the trial about a specific patient will be collected and stored electronically in the english language.

All data obtained will be kept under the supervision of the principal investigator who will be responsible for the safekeeping of the electronic data on site. In addition, as soon as practical, every new data generated will be scanned and e-mailed to the sponsor at the following addresses: mbazinet@replicor.com and availlant@replicor.com. This will be used to maintain a duplicate electronic copy of all patient data.

Principal investigator will be responsible for drug accountability on site.

5. INFORMED CONSENT

The principal investigator is responsible for ensuring that the patient understands the risks and benefits of participating in the study and should answer any questions the patient may have throughout the study in a timely manner. Also, the investigator must promptly inform the patient of important new developments that may impact the patient's willingness to continue participating in the study.

6. COSTS OF STUDY

All the costs incurred during the screening, surveillance, for inclusion and exclusion criteria during the selection process, the weekly laboratory evaluation and the fibroscan analysis will be covered by the sponsor. The drug will be provided for free for the duration of the trial.

7. REGULATORY CONSIDERATIONS

This study will be conducted in accordance with the ethical principles that are consistent with good clinical practice and the applicable laws and regulations of Bangladesh.

REP 102 Protocol Amendment:

Addition of thymosin alpha 1 (Zadaxin ™)
or Pegasys™ therapy in patients currently receiving
REP 2139 (REP 9AC') treatment

STUDY TITLE: Therapeutic safety and efficacy of REP 2139 (REP 9AC') in HBV infected patients.

PROTOCOL IDENTIFICATION NUMBER: REP 102

STUDY SPONSOR: REPLICor Inc., Laval, Canada

INSTITUTION: Bangabandhu Sheikh Mujib Medical Center, Dhaka, Bangladesh

STUDY STATUS: enrolment completed, all patients currently in treatment phase

STUDY DESIGN: Treatment, open label, safety / efficacy study, adaptive trial

ENROLLMENT: 12 patients

ESTIMATED STUDY START DATE: August 2011

STUDY AUTHORS: Mamun-Al-Mahtab, MD (Bangladesh), Principal Investigator
Andrew Vaillant, Ph.D. (REPLICor Inc.)
Michel Bazinet, MD (REPLICor Inc.)

1. INTRODUCTION

REP 9AC' (REP 2139) is a modified version of its predecessor, REP 9AC. Both are 40mer phosphorothioate oligonucleotides comprised of alternating adenosine and cytidine nucleotides and in the case of REP 9AC, has been shown to have low toxicity and to be highly effective in treating hepatitis B infection in human patients. The modifications in REP 9AC' significantly improve the stability and reduce the pro-inflammatory activity of REP 9AC' compared to that of REP 9AC while retaining all the antiviral activity found in REP 9AC. Both these modifications (5-methylation of cytosines and 2' O methylation of the ribose sugar in each nucleotide) are naturally occurring modifications in human nucleic acid and are known to be well tolerated in clinical trials.

Current interim data analysis from the still ongoing REP 102 protocol assessing the activity of REP 9AC’ in patients with chronic HBV infection indicates the following:

1. REP 9AC exposure in twelve patients has been generally well tolerated at doses up to 500mg / week. Chronic side effects include mild gastrointestinal discomfort (which is easily mitigated with a PPI such as omeprazole). The pro-inflammatory side effects observed with REP 9AC in the REP 101 protocol are almost absent with REP 9AC’ in the REP 102 protocol.

2. REP 9AC has achieved serum HBsAg reduction or clearance in 10 of 12 patients to date.

3. In these ten patients, all have demonstrated restoration of at least a partial immunological control of their infection (appearance of serum anti-HBs and the establishment of a lower serum HBV DNA setpoint).

4. In these ten patients, five have achieved a 4 – 6 log reduction in serum HBV DNA and are approaching the limit of quantification (116 copies/ml) of the assay.

5. In these five patients, the achievement of these serum HBV DNA reductions is correlated with a transient flare of serum ALT and AST, suggesting that restoration of the immune response following HBsAg reduction and or clearance is a key factor in controlling the infection. Similar ALT and AST flares were observed in patients achieving immunological control of their HBV infection in the REP 101 protocol.

It is now a widely held notion that any successful outcome in the treatment of chronic HBV must involve immunostimulation in order to catalyze restoration of the appropriate immune responses (both adaptive and innate) in order to achieve durable control over HBV infection after therapy. The results from the 19 patients who have experienced treatment with REP 9AC or REP 9AC’ clearly demonstrate that elimination of serum HBsAg removes the chronic immunosuppression mediated by this protein. However there is a heterogeneous immunological response to HBsAg reduction / clearance in the patients treated to date (as measured by serum HBV DNA decline): only about 40% of patients appear to exhibit a strong immunological response after the reduction / removal of serum HBsAg by REP 9AC / REP 9AC’. This observation strongly suggests that while HBsAg suppression is essential for establishing durable immunological control, specific immunostimulation may also be required in many patients in order for them to achieve durable immunological control while on REP 9AC / REP 9AC’ therapy.

2. Thymosin alpha as a potential add-on therapy for immunostimulation in patients currently on REP 2139 (REP 9AC) treatment.

Thymosin alpha 1 (sold as Zadaxin ™ by SciClone Pharmaceuticals) is currently approved as a monotherapy for the treatment of chronic HBV in the following countries:

Argentina, Azerbaijan, Bahrain, Brunei, Cambodia, China, Dominican Republic, Hong Kong, India, Indonesia, Kuwait, Kazakhstan, Kyrgyzstan, Laos, Malaysia, Maldives, Malta, Mexico, Moldova, Pakistan, Peru, Philippines, Russia, Singapore, Sri Lanka, Thailand, Ukraine, United Arab Emirates, Uzbekistan, Venezuela, Vietnam

Zadaxin™ is synthetically prepared thymosin alpha 1 polypeptide (28 amino acids, MW 3108) which is identical to the naturally occurring thymosin alpha 1 present in humans. Thymosin alpha 1 shares homology with the interferon alpha family of peptides but unlike interferon alpha, Zadaxin administration in human subjects is not accompanied by any significant side effects. More importantly, Zadaxin administration with other proinflammatory compounds (like interferon alpha) does not alter the side effect profiles of those compounds (1).

Zadaxin shares many of the immunostimulatory properties of interferon alpha in that it is able to stimulate the production of several cytokines important in reestablishing an immune response capable of controlling the HBV infection (1, 2). Zadaxin is also able to stimulate the production of NK, CD4 and CD8 cells all known to be correlated with the establishment of a durable immunological control of HBV infection (1, 2).

In the treatment of chronic HBV in the clinic, Zadaxin monotherapy is able to achieve HBeAg and HBV DNA seroclearance in 30-50% of patients which is comparable or better than that achievable with interferon alpha (1, 3, 4, 5, 6). More important is the consistent observation that the proportion of patients achieving HBV DNA seroclearance off therapy continually increases (an effect not observed with interferon alpha) (1).

Based on the very low side effect profile of Zadaxin (both as a monotherapy and in combination with interferon alpha and HBV polymerase inhibirors) and its clear ability to stimulate functional and durable immune response and control of HBV in patients with chronic HBV infections, the investigators strongly suspect that Zadaxin add-on therapy in patients currently receiving REP 9AC' will be safe and may have a **synergistic** effect on the ability of patients to achieve durable immunological control, thus greatly increasing the proportion of patients achieving durable immunological control with REP 9AC' – Zadaxin therapy compared to either therapy alone.

3. Pegasys ™ as a potential add-on therapy for immunostimulation in patients currently on REP 2139 (REP 9AC) treatment with no antiviral response on Zadaxin

It may be possible that the immunostimulation provided by Zadaxin™ may not provide a strong or broad enough immunostimulatory effect to provide an additive or synergistic antiviral response from the patient's immune system while on REP 9AC' therapy. Therefore, patients who tolerate Zadaxin™ / REP 9AC' combination therapy well but do not experience an improved or complete antiviral response after 8-10 weeks will be eligible to have the Zadaxin™ in their combination therapy replaced with Pegasys™. Since Pegasys™ is a much stronger immunostimulatory drug with significant side effects, dosing with this compound will start at 1/20th of the normal dose (9 ug once weekly) and slowly escalate each week to a full dose (180 ug once weekly) providing no grade 3 adverse events are observed. Pegasys™ exposure will not last more than 8-10 weeks in

the absence of any improved antiviral response compared to REP 9AC' alone and normally not exceed 24 weeks.

4. Proposed amendments to the REP 102 protocol.

Zadaxin™ combination therapy is to be implemented according to the following rules:

A. Early add-on rule: Patients not achieving serum HBV DNA < 2000 copies / ml after 20 weeks of REP 9AC' therapy who have not developed any persistent grade 3 or higher side effect are eligible to begin Zadaxin™ therapy. Zadaxin™ will be administered initially once weekly (1.6 mg SC injection) during the same visit in which patients receive their REP 9AC' by IV infusion. Zadaxin™ therapy can transition to twice weekly SC injections in the third week in the absence of any side effects grade 3 or higher.

B. Late add-on rule: Patients not achieving 4 consecutive weeks of HBV DNA < 200 copies / ml after 30 weeks of REP 9AC' treatment who have not developed any significant complications are eligible to begin Zadaxin™ therapy. Zadaxin™ will be administered initially once weekly (1.6 mg SC injection) which can then transition to twice weekly SC injections in the third week in the absence of any side effects grade 3 or higher.

C. Extension of combination REP 9AC' / Zadaxin™ therapy: Patients showing response to combination therapy (continued reduction in serum HBV DNA or achievement of serum HBV DNA < 116 copies / ml) may have their combination therapy extended beyond the current 40 week REP 9AC' limit (and 24 week Zadaxin™ limit) at the Principle investigator's discretion in order to achieve the goal of 4 continuous weeks of serum HBV DNA < 116 copies / ml prior to cessation of REP 9AC' therapy. This extension can only be permitted in patients who have not developed symptoms greater than grade 3 on combination therapy.

   Safety and efficacy monitoring will proceed without modification.

Pegasys ™ therapy is to be implemented according to the following rules:

A. Initiation of treatment: Patients who have had 8-10 weeks of Zadaxin™ / REP 9AC' combination therapy with no adverse effects grade 3 or greater and have had no significant improvement in their antiviral response compared to REP 9AC' alone and have no significant hematological or hepatic dysfunction are eligible to start REP 9AC / Pegasys ™ treatment.

B. Dose escalation: patients eligible to start REP 9AC / Pegasys™ combination treatment will receive an initial dose of 9ug of Pegasys™ (1/20th of the normally prescribed dose) by SC injection. Pegasys™ doses will escalate each week to 18ug, 45ug, 90ug and finally 180ug provided no adverse events grade 3 of higher are observed.

C. <u>Extension of combination REP 9AC' / Pegasys™ therapy</u>: Patients showing response to combination therapy (continued reduction in serum HBV DNA or achievement of serum HBV DNA < 116 copies / ml) may have their combination therapy extended beyond the current 40 week REP 9AC' limit (and 24 week Pegasys™ limit) at the Principle investigator's discretion in order to achieve the goal of 4 continuous weeks of serum HBV DNA < 116 copies / ml prior to cessation of REP 9AC' therapy. This extension can only be permitted in patients who have not experiences any adverse events greater than grade 3 on combination therapy.

<u>Hematology will be monitored on a weekly basis while patients are on REP 9AC' / Pegasys™ combination therapy.</u>

References (attached).

1. Zadaxin drug monograph from SciClone pharmaceuticals.
2. Jiang et al. Journal of Int. Med. Res. 2010; 38: 2053-2062.
3. Iino et al. J. Viral Hepatitis 2005; 12: 300-306.
4. Andreone et al. Hepatology 1996; 24: 774-777.
5. Zhuang et al. World J. Gasterenterol. 2001; 7: 407-410.
6. You et al. World J. Gasterenterol. 2006; 12: 6715-6721.
