# Supplementary material for: Safety and Efficacy of Nucleic Acid Polymers in Monotherapy and Combined with Immunotherapy in Treatment-Naive Bangladeshi Patients with HBeAg+ Chronic Hepatitis B Infection
Source: PLoS One. 2016 Jun 3;11(6):e0156667. doi: 10.1371/journal.pone.0156667 (PMC4892580; doi:10.1371/journal.pone.0156667)
Supplement: S1 Table — (DOCX) [file pone.0156667.s004.docx]

Supplementary Table 1: Ishak and Knodell scores from pre-treatment liver biopsies in REP 101 patients.

| Patient | Ishak modified hepato-activity index score | Knodell Score |
| --- | --- | --- |
| 1 | 12 | 16 |
| 2 | 11 | 14 |
| 3 | 7 | 8 |
| 4 | 3 | 1 |
| 5 | 9 | 3 |
| 6 | 9 | 10 |
| 7 | 3 | 3 |
| 8 | 5 | 6 |
