# Supplementary material for: Safety and Efficacy of Nucleic Acid Polymers in Monotherapy and Combined with Immunotherapy in Treatment-Naive Bangladeshi Patients with HBeAg+ Chronic Hepatitis B Infection
Source: PLoS One. 2016 Jun 3;11(6):e0156667. doi: 10.1371/journal.pone.0156667 (PMC4892580; doi:10.1371/journal.pone.0156667)
Supplement: S2 Table — (DOCX) [file pone.0156667.s005.docx]

Supplementary Table 2: IV infusion adverse reactions in the REP 101 study.

| **IV infusion adverse events (> 10h infusion) with prophylactic anti-histamine** | **Cumulative Incidence from 322 infusions** | **% occurrence** |
| --- | --- | --- |
| Fever | 21 | 6.52 |
| Itchy palms / soles / eyes ^a^ | 17 | 5.28 |
| Rigor | 11 | 3.42 |
| Hyperemia eyes / face ^a^ | 6 | 1.86 |
| Increased effort to breathe | 6 | 1.86 |
| Nausea | 5 | 1.55 |
| Chills | 4 | 1.24 |
| Body ache | 3 | 0.93 |
| Weakness | 3 | 0.93 |
| Vomiting | 3 | 0.93 |
| Burning sensation in eyes | 1 | 0.31 |
| Watery eyes | 1 | 0.31 |

^a^ considered related to the use of DEHP containing IV tubing.
