# Supplementary material for: Safety and Efficacy of Nucleic Acid Polymers in Monotherapy and Combined with Immunotherapy in Treatment-Naive Bangladeshi Patients with HBeAg+ Chronic Hepatitis B Infection
Source: PLoS One. 2016 Jun 3;11(6):e0156667. doi: 10.1371/journal.pone.0156667 (PMC4892580; doi:10.1371/journal.pone.0156667)
Supplement: S3 Table — (DOCX) [file pone.0156667.s006.docx]

Supplementary Table 3: Treatment related adverse events in the REP 101 study.

| **Adverse Event** | **Reported Incidence in 8 patients (REP 2055 treatment 20-55 weeks)** |
| --- | --- |
| Weakness | 12 |
| Fever | 4 |
| Reduced appetite | 4 |
| Reduced sleep | 2 |
| Restlessness | 2 |
| Gum bleeding | 2 |
| Loose stool | 2 |
| Abdominal distension | 2 |
| Hypocalcemia | 2 |
| Epigastric pain | 2 |
| Body ache | 2 |
| Intercostal pain with breathing | 2 |
| Tingling sensation in extremities | 1 |
| Dyspepsia | 1 |
| Elevated serum bilirubin (self-resolving) | 1 |
| Thrombocytopenia | 1 |
| Burning sensation in scalp | 1 |
