# Supplementary material for: Safety and Efficacy of Nucleic Acid Polymers in Monotherapy and Combined with Immunotherapy in Treatment-Naive Bangladeshi Patients with HBeAg+ Chronic Hepatitis B Infection
Source: PLoS One. 2016 Jun 3;11(6):e0156667. doi: 10.1371/journal.pone.0156667 (PMC4892580; doi:10.1371/journal.pone.0156667)
Supplement: S4 Table — (DOCX) [file pone.0156667.s007.docx]

Supplementary Table 4: IV infusion adverse reactions in the REP 102 study.

| **IV infusion adverse event (2h infusion), no supportive therapy** | **Cumulative incidence (from 525 infusions)** | **% occurrence** |
| --- | --- | --- |
| Fever | 40 | 7.62 |
| Shivering | 22 | 4.19 |
| Shivering | 22 | 4.19 |
| Chills | 8 | 1.52 |
| Body ache / cramping | 8 | 1.52 |
| Headache | 6 | 1.14 |
| Vomiting | 4 | 0.76 |
| Dizziness / vertigo | 3 | 0.57 |
| Itchy or tingling palms / soles / eyes | 3 | 0.57 |
| Increased effort to breathe | 2 | 0.38 |
| Generalized itching | 1 | 0.19 |
| Hypertension | 1 | 0.19 |
