# Supplementary material for: Safety and Efficacy of Nucleic Acid Polymers in Monotherapy and Combined with Immunotherapy in Treatment-Naive Bangladeshi Patients with HBeAg+ Chronic Hepatitis B Infection
Source: PLoS One. 2016 Jun 3;11(6):e0156667. doi: 10.1371/journal.pone.0156667 (PMC4892580; doi:10.1371/journal.pone.0156667)
Supplement: S5 Table — (DOCX) [file pone.0156667.s008.docx]

Supplementary Table 5 – Treatment related adverse events in the REP 102 study

| **Adverse Event** | **Reported Incidence** | |
| --- | --- | --- |
|  | 12 patients REP 2139-Ca  (20-37 weeks) | 5 patients REP 2139-Ca + Immunotherapy |
| Dyspepsia | 18 | 28 |
| Reduced appetite | 15 | 23 |
| Fever | 14 | 5 |
| Weakness | 13 | 19 |
| Loose stool / increased frequency of BM ^a^ | 9 | 5 |
| Generalized body ache | 9 | 19 |
| Pain, tingling or lack of sensation in extremities | 7 | 5 |
| Back pain | 8 | 5 |
| Epigastric pain | 6 | 3 |
| Generalized itching / rash | 6 | 4 |
| Hair loss ^b^ | 5 | 7 |
| Headache | 5 | 1 |
| Joint pain / arthralgia | 4 | 11 |
| Abdominal cramping / pain | 2 | 3 |
| Nausea / vomiting | 2 | 4 |
| Dizzyness / Vertigo | 2 |  |
| Muscle cramping / pain | 1 | 2 |
| Conjunctival hyperemia | 1 | 2 |
| Hemophysis | 1 |  |
| Blurred vision | 1 |  |
| Acid reflux | 1 |  |
| Depression | 1 |  |
| Flank Pain (rt. side) | 1 | 3 |
| Heart palpitations | 1 |  |
| Loose teeth / toothache |  | 4 |
| Dysphagia ^b^ |  | 9 |
| Dysgeusia ^b^ |  | 1 |
| Weight loss |  | 5 |
| Hyperemia of palms or soles |  | 3 |
| Bleeding / swollen / painful gums |  | 3 |
| Reduced sleep |  | 2 |

^a^ attributed to magnesium supplementation.

^b^ present in all patients during follow-up
